# Nested pool testing strategy for the diagnosis of infectious diseases

**DOI:** 10.1038/s41598-021-97534-7

**Published:** 2021-09-13

**Authors:** Inés Armendáriz, Pablo A. Ferrari, Daniel Fraiman, José M. Martínez, Hugo G. Menzella, Silvina Ponce Dawson

**Affiliations:** 1 Departamento de Matemática & IMAS, FCEN-UBA & CONICET, C1428EGA Buenos Aires, Argentina; 2 Departamento de Matemática y Ciencias, Universidad de San Andrés & CONICET, B1644BID Victoria, Argentina; 3 Department of Applied Mathematics, IMECC-UNICAMP, Campinas, 13083-859 Brazil; 4 Departamento de Tecnología & IPROByQ, FBIOyF-UNR & CONICET, S2002LRK Rosario, Argentina; 5 Departamento de Física & IFIBA, FCEN-UBA & CONICET, C1428EGA Buenos Aires, Argentina

**Keywords:** Mathematics and computing, Epidemiology, Population screening, High-throughput screening, Assay systems

## Abstract

The progress of the SARS-CoV-2 pandemic requires the design of large-scale, cost-effective testing programs. Pooling samples provides a solution if the tests are sensitive enough. In this regard, the use of the gold standard, RT-qPCR, raises some concerns. Recently, droplet digital PCR (ddPCR) was shown to be 10–100 times more sensitive than RT-qPCR, making it more suitable for pooling. Furthermore, ddPCR quantifies the RNA content directly, a feature that, as we show, can be used to identify nonviable samples in pools. Cost-effective strategies require the definition of efficient deconvolution and re-testing procedures. In this paper we analyze the practical implementation of an efficient hierarchical pooling strategy for which we have recently derived the optimal, determining the best ways to proceed when there are impediments for the use of the absolute optimum or when multiple pools are tested simultaneously and there are restrictions on the throughput time. We also show how the ddPCR RNA quantification and the nested nature of the strategy can be combined to perform self-consistency tests for a better identification of infected individuals and nonviable samples. The studies are useful to those considering pool testing for the identification of infected individuals.

## Introduction

The progress of the SARS-CoV-2 pandemic still requires the implementation of large-scale testing programs. Although vaccination might bring a relief in some countries, the widespread availability of vaccines in the developing world will take quite some time. Viral mutations, on the other hand, might also affect the effectivity of vaccination plans. Diagnostic tests are required for the analysis of symptomatic individuals and for the early detection of asymptomatic or pre-symptomatic carriers. They are a powerful tool used in surveillance at sites of previous or potential outbreaks and for environmental monitoring. Massive testing campaigns demand the maximum efficiency in the use of the available resources^1^. Mixing (*pooling*) multiple samples to test them in a single reaction is an option to increase testing capacity reducing costs and time. The first proposal of a pooling strategy to detect infected individuals^2^ was subsequently improved to further reduce costs^3–7^, to simplify its practical implementation^8^ or to correct for limitations in detectatibility^9–14^. In a separate paper^15^ we compute the optimal strategy for the scheme^9,10^, assuming no testing errors. Various pool testing strategies have been analyzed specifically for the case of SARS-CoV-2^16–20^ (see Supplementary Note for more details). Despite the initial excitement, it is accepted that in RT-qPCR the target can go unidentified if present in small amounts^21^. Diagnostic sensitivity in symptomatic patients was reported to be in the range of 60%–90%^22^. Thus, the pooling associated dilution could further reduce the detection of infected individuals to unacceptable levels. Recently, great improvements in the sensitivity and specificity of SARS-CoV-2 RNA detection have been introduced by using droplet digital PCR (ddPCR)^23–25^. In ddPCR the sample is emulsified with oil to split the reaction in thousands of droplets^26^. Each droplet works as a tiny reactor, reducing the chance of interferences and favoring the detection of even a single molecule of the target nucleic acid. A direct comparison in samples with low RNA concentration and variable amounts of inhibitors, ddPCR was shown to be up to 500 times more sensitive than RT-qPCR^21,27^. The use of ddPCR or of other technologies with increased sensitivity like Next Generation Sequencing^28^ would remove therefore the major barrier for the adoption of group testing strategies, raising the need to rapidly obtain efficient algorithms for pool testing^29^. Here we consider the family of adaptive hierarchical strategies introduced in^9^, and address different issues that may arise in the practical implementation of the optimal strategy in this family^15^, when applied to identifying virally infected individuals using ddPCR.

The strategies in this family are multistage extensions of Dorfman’s algorithm^9,10^ that have simple analytic expressions for the cost as a function of the infection probability, *p*. This allowed us to derive the optimum choice of strategy in the family as a function of *p*^15^. Here we analyze how to proceed when *p* is not known a priori, when there are constraints that prevent the optimal strategy from being used or when the strategy is applied in parallel on several pools simultaneously and there are restrictions on the throughput time for logistic reasons. One of the problems that are inherent to pool testing is the level of reliability with which an individual can be identified as not infected when its sample has not been tested individually. In this paper we show how the use of ddPCR can provide a realiable such identification thanks to the quantification of the RNA content that the technique gives at the end of the test. We also show how the combination of this quantification capability with the nested nature of the strategy can be used for self-verification purposes. Finally, we use the quantification formulas of ddPCR to estimate the probability with which the presence of a single infected sample in a pool may be detected as a function of the viral load and pool size and analyze the sensitivty of our method. Regarding cost reduction, for small enough *p*, the expected number of tests per individual of our optimal strategy is^15^ $\sim 3p \log (1/p)/\log (3)\approx 2.7p \log (1/p)$, which is similar to that of the hypercube testing strategy of^30^ and slightly larger than that of the mixed strategy of^7^, $\sim p\log (1/p)/\log ^2(2)\approx 2.1p\log (1/p)$ or of the binary splitting^5^ one, $\sim p\log (1/p)/\log (2)\approx 1.4 p\log (1/p)$. The cost reduction of the latter, however, is achieved at the expense of having an unknown number of stages which is impractical for clinical laboratories due to the pressure imposed by fixed delivery time frames. The simplicity of our optimal strategy and the advantages of its nested nature make it a very valuable option to faithfully detect viral genomes saving costs at the same time. We think it can be very useful to minimize the variable costs of SARS-CoV-2-carrier detection and optimize the use of resources in testing facilities. An interface for interested users of the method is available at^31^.

## Results and discussion

### Presentation of the strategy

**Figure 1:**
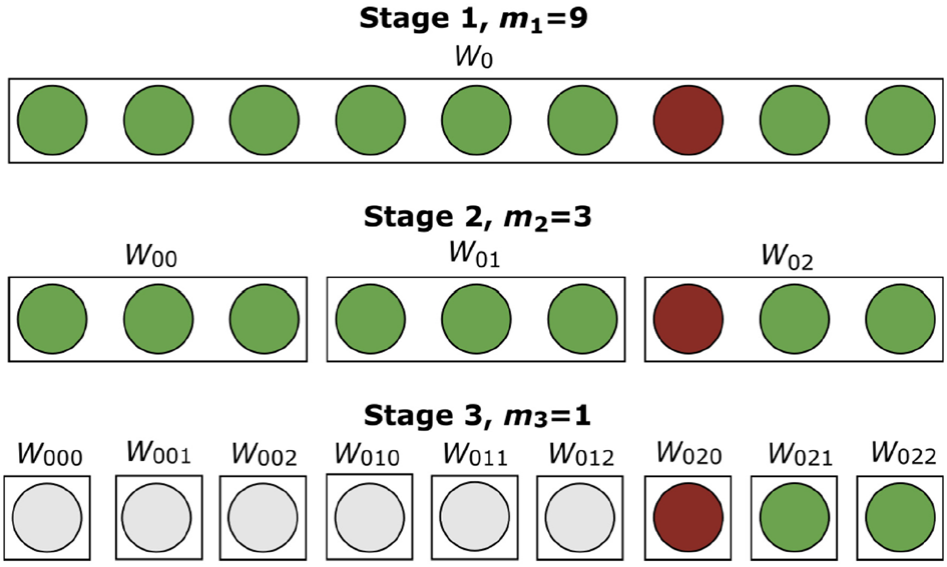
Schematic depiction of the nested pool testing strategy with $k=2$ and $m=(9,3)$ when applied to one pool labeled, $W_0$. The circles represent individual samples and the rectangles represent pools. The red circles correspond to infected samples. The non-infected circles are either green (when they belong to a pool that is being tested at the corresponding stage) or grey (when they have been identified as non-infected at a previous stage). At the first stage all 9 samples of the pool, $W_0$, are tested together. The result is positive due to the presence of one infected sample. $W_0$ is then divided into 3 sub-pools, $W_{00}$, $W_{01}$, $W_{02}$, of 3 samples each that are tested at the second stage. After these tests, $W_{00}$ and $W_{01}$, are identified as not infected so that the samples in them are not further tested. $W_{02}$ is identified as infected and all its samples are tested individually at the last stage. In the illustration the volume occupied by each pool seems to decrease from stage to stage: the pool volume is actually the same at all stages. The figure illustrates the labels, $W_{i_1...i_j}$, that can be assigned *a priori* to individual samples and pools of all stages, regardless of whether they are going to be tested as such or not. The labeling is characterized by a sequence of as many subscripts, ${i_1,\ldots ,i_j}$, as stage number with, $i_1$, the subscript that identifies the initial pool (in the figure, $i_1=0$).

The strategy^9,10^ that we consider in the present paper is illustrated in Fig. 1. For its definition and optimization we assume that the test detects the presence of a single infected sample in a pool. We analyze later how to assess the occurrence of false negatives when testing pools. The strategy is characterized by the number of stages, $$(k+1)$$, and the sequence of pool sizes, $$m=(m_1,\ldots ,m_k)$$, where $$m_1>\dots>m_k>1$$ and $$m_j $$ is a multiple of $$m_{j+1}$$ for all *j*. At the first stage, pools with $$m_1$$ samples are tested. The samples in each of those that test positive are combined in pools of size $$m_2$$ following a nested scheme (see Fig. 1) and are tested at a second stage. The procedure is repeated until the $$k+1$$-th stage at which all the samples contained in pools that tested positive at the *k*-th stage are tested individually, i.e., $$m_{k+1}=1$$.

**Figure 2:**
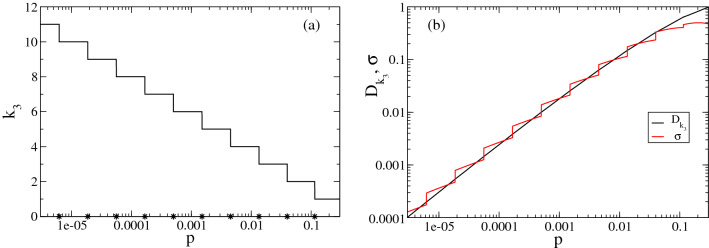
Properties of the optimal nested strategy, $m=(3^k,\ldots ,3)$, as functions of *p*. (**a**) The number of stages, $k=k_3$, with $k_3$ given by Eq. (13) and $\mu = 3$, is a piece-wise constant decreasing function of *p* with discontinuities at the probabilities indicated with stars on the horizontal axis. (**b**) The expected value, $D_{k_3}(m, p)$ (black curve), and standard deviation, $\sigma (m,p)$ (red curve), of the number of tests per individual stay close to one another and increase monotonically with *p*.

The cost of the strategy, $D_k(m,p)$, is defined as the expected number of tests per individual, $ET_k/m_1$, for which there is an analytic expression^9,10,12,14^ as a function of the infection probability, *p* (see Methods). For each *p*, $D_k(m,p)$ is minimized by the optimal strategy^15^. For all but a tiny set of values of *p*, the optimal has $m=(3^k,\ldots ,3)$ with $k=k_3(p)$ given by Eq. (13) (with $\mu =3$)^15^. For the values of *p* for which it is not the optimum, its cost differs by a negligible amount with respect to the optimum (see Supplementary Note). We show in Fig. 2 the number of stages, $k_3(p)$, in (a) and the expected number of tests per individual, $D_k$ (black, Eq. 12), and standard deviation of this number $\sigma (m,p)$ (red, Eq. (14)), in (b), for the $m=(3^{k_3(p)},\ldots ,3)$ strategy. $k_3(p)$ increases with decreasing *p*. Thus, it only makes sense to use it if^15^:

1 $$\begin{aligned} p<1-1/3^{1/3} \approx 0.3066387\dots , \end{aligned}$$

which is the equivalent of the upper bound of Dorfman’s for integer-sized pools.

### Expected cost and variability

The optimization problem worked out in^15^ looked at the minimization of the expected value of the number of tests per individual. Any realization of the strategy, however, will result in a number of tests that will differ from the expected value. Although the repeated application of the strategy will approach, on average, the expected cost, it is important to assess the variability around the mean. As shown in Fig. 2b the standard deviation of the number of tests per individual of the optimal strategy is of the same order of magnitude as the expected value. In this Section we present the results of stochastic numerical simulations, performed as explained in Methods, to understand the nature of this variability showing that it does not yield increasing costs.

**Figure 3:**
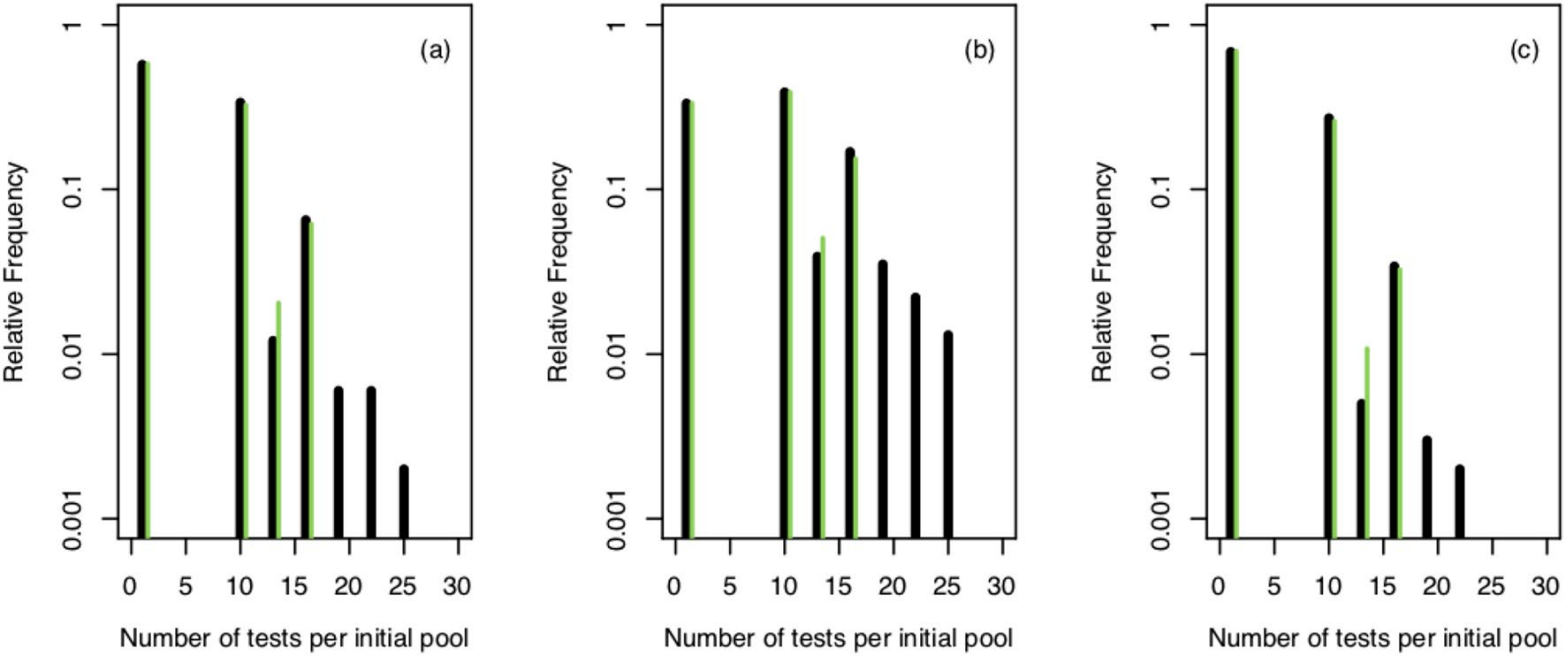
Stochastic simulations of the nested strategy with $k=3$ and $m=(3^3,3^2,3)$ applied to 1000 first-stage pools with infection probabilities, *p*, for which the strategy is optimal (the one in (**b**), slightly smaller than the value at which the optimum switches from having $k=2$ to having $k=3$ and the one in (**c**), slightly larger than the value at which the optimum switches from having $k=3$ to having $k=4$). The figures show histograms (as fraction of occurrences and in log scale) of the number of tests performed per pool. (**a**) $p=0.02$ (544 infected samples, prevalence $\overline{p}=0.0201$) (**b**) $p=0.0398$ (1073 infected samples, prevalence $\overline{p}=0.0201$) (**c**) $p=0.0135$ (369 infected samples, prevalence $\overline{p}=0.0201$). Green bars correspond to the theoretical values that we computed (see Supplementary Note). The apparent mismatch between theory and simulations is solely due to fluctuations and disappears as the number of initial pools is increased.

We show in Fig. 3 histograms of the number of tests performed per pool obtained from simulations of the strategy with $k=3$ and $m=(3^3,3^2, 3)$ for values, *p*, for which it is optimal. 1000 realizations (initial pools) were done for each *p*. In Fig. 3a approximately 58% of the 27-sample pools required only one test, i.e., under ideal conditions, 15,660 individuals could be reported as negative using only 580 tests. This is key for the large reduction in the average number of tests per individual ($\sim 0.2$, instead of 1) and per pool ($\sim 5.4$ instead of 27) of the example. The second most frequent situation in Fig. 3a, with $\sim 34\%$ of the instances, is the one with 10 tests/pool. This corresponds to initial pools with only one infected sample or with more that remain in the same pool until the *k*-th stage, for which the total number of tests and the number of tests per individual are, respectively (see Supplementary Note):

2 $$\begin{aligned} {\hat{T}}_k&=1+m_k +\sum _{j=2}^k \frac{m_{j-1}}{m_j}=1+k\mu , \end{aligned}$$

3 $$\begin{aligned} {\overline{D}}_k(m_1,\ldots , m_k,\tilde{p})&=\frac{1}{m_1}+m_k \tilde{p}+\tilde{p}\sum _{j=2}^k \frac{m_{j-1}}{m_j}, \end{aligned}$$

with $\tilde{p}=1/m_1$. The comparison of the three examples of Fig. 3 provides an explanation for the relationship between the cost and standard deviation displayed in Fig. 2b. We observe in Fig. 3 that, as *p* decreases within the range of optimality of the strategy with $k=3$ and $m=(3^3,\ldots ,3)$ the fraction of initial pools that require the largest number of tests decreases while the fraction of those that require only one test (i.e., no infected samples), grows. The increasing ratio, $\sigma /D_3$, with decreasing *p* that is apparent in Fig. 2b can then be related to an increasing fraction of instances with no infected samples that are resolved with only one test. This is easy to explain if we assume that the pools can have at most one infected sample. In such a case, those with no infected samples are solved with a single test while the others require a total number of tests, ${\hat{T}}_k$, given by Eq. (2). Defining the stochastic variable, *X*, so that $X=0$ if the pool contains no infected samples and $X=1$ otherwise and calling, ${\hat{p}}$, the probability that $X=1$, the mean and standard deviation of *X* are $\langle X\rangle = {\hat{p}}$ and ${\hat{\sigma }}= \sqrt{{\hat{p}}(1-{\hat{p}})}$ and satisfy ${\hat{\sigma }}/\langle X\rangle >1$ for ${\hat{p}}<1/2$ with ${\hat{\sigma }}/\langle X\rangle$ increasing for decreasing ${\hat{p}}<1/2$. Thus, the ratio increases because the deviation approaches zero more slowly than the mean. The “worst” pools (those with exactly one infected sample) are solved with a number of tests given by Eq. (2), which is smaller than the number of samples in the initial pool, $\mu ^k$, for $\mu =3$ and $k\ge 2$. Thus, having a standard deviation of the same order of magnitude as the cost is reflecting the relatively high chance that the number of tests to perform will be smaller than the expected value.

### Practical implementation of the nested strategy: constraints and runs in parallel

In this Section we present a series of analyses to determine the best way to proceed when there are constraints that prevent the use of the theoretically optimal nested strategy or when the strategy is applied in parallel on various pools simultaneously.

#### Pool testing under unknown prevalence

The infection prevalence of the analyzed population can be estimated as explained in the Supplementary Note to determine the optimal strategy.

**Figure 4:**
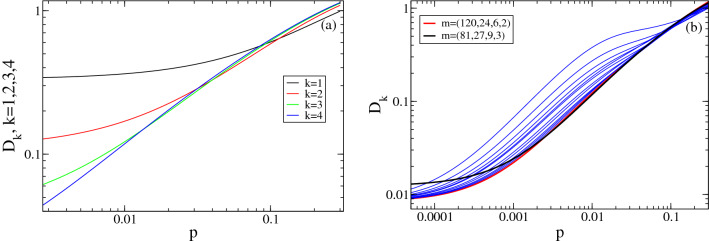
Cost of different nested strategies. (**a**) $D_k(m,p)$ *vs* *p* for strategies with $m=(3^k,\ldots ,3)$ and $k=1$ (black), $k=2$ (red), $k=3$ (green), $k=4$ (blue). (**b**) $D_k(m,p)$ *vs* *p* for the strategies with $m=(120,20,4)$, $m=(120,15,3)$, $m=(120,12,3)$, $m=(120,10,2)$, $m=(120,8,2)$, $m=(120,6,2)$, $m=(120,6)$, $m=(120,5)$, $m=(120,4)$, $m=(120,3)$ and (120, 2) (blue), $m=(120,24,6,2)$ (red) and $m=(81, 27 , 9,3)$ (black).

We analyze in this subsection the cost of applying the strategy, $(3^k,\ldots ,3)$ with $k=k_3(p)$ to cases with infection probability different from *p*. We compare in Fig. 4 the cost, $D_k(m,p)$, of the strategies with $m=(3^k,\ldots ,3)$ and different values of *k* for all values of *p*. Let us call $(p_{3,k+1},p_{3,k})$ the interval of optimality of the strategy with $k+1$ stages (the interval where $k_3(p)=k$). At $p=p_{3,k+1}$, it is $D_k=D_{k+1}$, with $D_k<D_{k+1}$ for $p>p_{3,k+1}$ and $D_k\ge D_{k+1}$, otherwise. We observe that, even if not optimal, the $(k+1)-$th stage strategy produces a noticeable reduction in the cost for $p<p_{3,k+1}$. This is related to the results of Fig. 3: when a strategy with $m=(3^k,\ldots ,3)$ is applied to situations with $p<p_{3,k+1}$, initial pools with more than one infected sample become increasingly rare and the cost is correctly approximated by Eq. (3), which is smaller than 1. The procedure to estimate *p* as the strategy is applied that is described in the Supplementary Note is based on this analysis.

*Based on this discussion we conclude that, if the value ofpis poorly known a priori, it is advisable to use a strategy with*$m=(3^k,\ldots ,3)$*and a relatively small number of stages,*$(k+1)$. As discussed in the Supplementary Note, after the strategy is applied to several pools, the estimate of *p* can be improved and the value of *k* be retuned.

#### Maximum number of samples allowed in a pool

We now analyze how to proceed if the optimal strategy requires a value, $m_1$, that is larger than the maximum allowed for the test to be reliable. We arbitrarily set the bound $m_1\le 120$ and assume that *p* is such that the optimal $m=(3^k,\ldots ,3)$ strategy has $k+1=6$ and $m_1=3^5=243>120$ ($p_{3,6}\approx 0.0015059<p <0.0045108\approx p_{3,5}$). As explained in the Supplementary Note, the constrained optimization problem can be solved algorithmically more efficiently using some analytic results of^15^. In particular, if $m_1=120$ the strategies whose costs should be compared to decide the optimal are: $m=(120,24,6,2)$, $m=(120,20,4)$, $m=(120,15,3)$, $m=(120,12,3)$, $m=(120,10,2)$, $m=(120,8,2)$, $m=(120,6,2)$, $m=(120,6)$, $m=(120,5)$, $m=(120,4)$, $m=(120,3)$ and (120, 2). We show in Fig. 4b these strategies and the strategy of the form $m=(3^k,\ldots ,3)$ with the largest *k* such that $m_1=3^k<120$. We observe that the $m=(3^4,3^3,3^2,3)$ scheme has the smallest cost even for values, $p< p_{3,5}\approx 0.0045$, outside its region of optimality and that its cost keeps on reducing as *p* is decreased. For the smallest values of *p*, the strategy with $m=(120,24,6,2)$ is best. As *p* is reduced beyond the values displayed in Fig. 4b, the costs of all the strategies with $m_1=120$ approach the same value, which is smaller than the cost approached by the $m=(3^4,3^3,3^2,3)$ strategy. For small enough *p*, there is at most one infected sample per pool, the expected number of tests per individual is given by Eq. (3) with $\tilde{p}=p$ and is dominated by the term, $1/m_1$. Thus, the larger $m_1$, the larger the reduction.

*We then conclude that, given an upper bound,*$m_{1, max}$, *on the number of samples that can be combined in a pool, and an infection probability,p*, *such that*$3^{k_3(p)}>m_{1, max}$*it is advisable to use the strategy,*$m=(3^k,\ldots , 3)$, *with*$k = \lfloor {\log _3(m_{1,max})}\rfloor$, *ifpis not much smaller than the probability for which the strategy is optimal. For very smallp*, *larger reductions are achieved as*$m_1$*grows, regardless of whether*$m_1= 3^k$*or not. The search for the optimal strategy can be done algorithmically as described in the Supplementary Note. Starting with*$m_1= 3^k$, *however, has other advantages as discussed in the following.*

#### Running the nested strategy in parallel

**Figure 5:**
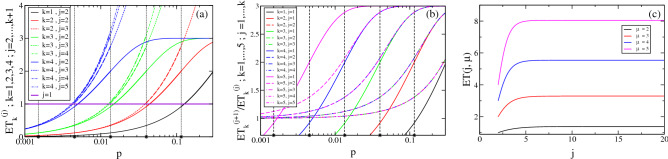
Expected number of tests per stage. (**a**) Expected number of tests, $E{T}^{(j)}_{k}(m,p)$, at each stage, *j*, with $2\le j\le k$, as a function of *p* for strategies of the form $m=(3^k,\ldots ,3)$ with $k=1$ (black), $k=2$ (red), $k=3$ (green) and $k=4$ (blue). The thicker portion of the curves correspond to the values of *p* for which each of the schemes is optimal. The horizontal line in magenta corresponds to the number of tests at the first stage ($j=1$) for all *k*. (**b**) Ratio of the number of tests expected for two subsequent stages, $E{T}^{(j+1)}_{k}(m,p)/E{T}^{(j)}_{k}(m,p)$, of the strategies with $m=(3^k,\ldots ,3)$ and $k=1$ (black), $k=2$ (red), $k=3$ (green), $k=4$ (blue) and $k=5$ (magenta). (**c**) $ET(j,\mu )=E{T}^{(j)}_{k}(\mu ^{k_\mu },\ldots ,\mu ,p_k)$, as a function of *j* for $\mu =2$ (black), $\mu =3$ (red), $\mu =4$ (blue) and $\mu =5$ (magenta). We plotted the curves for any value of *j*, but only integer ones are meaningful.

For testing facilities, cost reduction is not only achieved through savings in reagents but also by reducing the throughput time. For the latter, PCR tests are usually run on various sets simultaneously using multi-well plates. If our strategy were applied in such a way that all the tests were performed sequentially, the expected throughput time would be proportional to the expected number of tests. If the strategy is run in parallel on a fixed number of simultaneous pools, this relationship between throughput time and number of tests no longer holds. Facilities face the pressure to deliver their results on a reliable time frame. For this, it is important that they have an estimate of the expected throughput time or, equivalently, the number of samples processable per unit time. In the case of a pandemic like the SARS-CoV-2 one, it is also important for governments and other institutions to decide the most efficient scheme to test their populations of concern. Coming back to our strategy, suppose that, at the first stage, the test is run on as many pools as the number of wells in the plates available in the facility, i.e., all the wells are used. The question then arises of what happens at subsequent stages. If more wells are needed than those available, then the application of the strategy will require additional rounds of tests. In this section we analyze this aspect. To this end, let us assume that we use a $k+1$ nested strategy with pool sizes $m=(m_1,\ldots ,m_k)$ in a set-up that allows $N_w$ tests to be performed in parallel. Let us assume that, at the first stage, we use all the available wells, i.e., we run the test on $N_w$ pools. We want to compare the number of pools to be tested at subsequent stages with $N_w$. This is equivalent to comparing the number of tests, $ET^{(j)}_k$ (Eq. (11)), that are expected to be performed at each stage, *j*, of the strategy, $m=(m_1,\ldots ,m_k)$, starting from one initial pool, with the number one. We show in Fig. 5a a plot of $E{T}^{(j)}_{k}(m,p)$, $2\le j\le k$, as a function of *p* for strategies with $m=(3^k,\ldots ,3)$ and various, *k*. The curves are drawn with thicker lines over the range of optimality of each scheme. The horizontal line (magenta) corresponds to the first stage (always equal to 1). We observe that $E{T}^{(j)}_{k}(m,p)$ increases with *p* for every *j* and *k*. Thus, within its range of optimality, $(p_{3,k_3+1},p_{3,k_3})$, the $m=(3^{k_3}, \ldots , 3)$ scheme requires the largest number of tests (on average) for $p=p_{3,k_3}$. The number of tests expected at the second stage ($j=2$) is about twice the number at $j=1$ for $p=p_{3,k_3}$ while both numbers are approximately equal for $p_{3,k_3+1}$. For fixed *k* and *p*, $E{T}^{(j)}_{k}$ increases with *j*. Thus, we can expect the largest number of tests at the last stage, $k+1$. The ratio of the number expected for two subsequent stages, however, never exceeds $\sim 2.1$ for any, *p*, smaller than the maximum value for which the strategy is optimal. This is reflected in Fig. 5b where we have plotted the ratio $E{T}^{(j+1)}_{k}(m,p)/E{T}^{(j)}_{k}(m,p)$ with $m=(3^k,\ldots ,3)$ and $1\le j\le k$ for various *k*. All the ratios decrease with decreasing *p*, those with $j\ge 2$ approach 1 as $p\rightarrow 0$ with an unnoticeable *k* dependence and the ratio, $E{T}^{(2)}_{k}/E{T}^{(1)}_{k}$, goes to zero (as $m_1^2p/m_2$). Computing $E{T}^{(j)}_{k}(m,p)$ for $m=(\mu ^{k},\ldots ,\mu )$ at the highest end of its region of optimality, $p=p_{\mu ,k_\mu }$, we obtain:

4 $$\begin{aligned} E{T}(j,\mu )\equiv E{T}^{(j)}_{k}(\mu ^{k_\mu },\ldots ,\mu ,p_k)&=\mu ^{j-1} \left( 1-\mu ^{-\mu ^{2-j}}\right) \qquad 2\le j \le k+1, \end{aligned}$$

which is independent of *k*. We show in Fig. 5c plots of $E{T}(j,\mu )$ as functions of *j* for various values of $\mu$. We observe that $E{T}(j,\mu )$ increases with *j* approaching the limiting value, ${\mathbf{ET_\mu }}= \mu \log (\mu )$. This implies that, if we use a $m=(3^{k_3},\ldots ,3)$ scheme for situations with $p\le p_{3,k_3}$, we can expect that the number of tests at each stage will never exceed ${\mathbf{ET_3}}\sim 3.296$ times the number of tests of the first stage. The upper bound, ${\mathbf{ET_\mu }}$, increases with $\mu$. Thus, the largest $\mu$, the largest will be the expected number of tests at any given stage compared with the first one (given that $p\le p_{\mu ,k}$). This is important when running the strategy in parallel on $N_w$ pools: having to test more pools than $N_w$ (what we call *overflow*) at stages $j\ge 2$ complicates the book-keeping and increases the throughput time. Table 1 illustrates how the strategy $m=(27,9,3)$ performs for $p=0.02$ when run on 96 pools simultaneously. In this table, the time unit identifes the number of the run. Given that up to 96 wells can be tested simultaneously in this example, in principle, new pools of samples could be accommodated in the empty wells of runs 5–7. The optimization in the use of these wells will be the matter of future studies. This and other examples are compared in greater detail in the Supplementary Note. The interface available at^31^ not only gives information on the expected cost but also on the throughput time for users to choose, among the various strategies they probed, which one best meets their needs.

**Table 1:** Running the $m=(27,9,3)$ strategy in parallel on 96 pools per time unit for an example with 2592 samples (53 infected). Only after the first 4 time units, it is possible to identify some infected individuals. In the example, the remaining infected individuals are identified at the end. This feature is highlighted in bold in the table by quoting the total number of cases informed after the 4th time unit and at the end.

Time unit	Pool size	# of tests	Infected pools detected	Pool overflow	Identified samples	
					Not-infected	Infected
1	27	96	39	0	1539	0
2	9	96	41	21	495	0
3	3	96	35	27	183	0
4	1	96	32	9	64	32
**Accumulated number of cases informed**					**2281**	**32**
5	9	21	8	0	117	0
6	3	51	18	0	99	0
7	1	63	21	0	42	21
**Accumulated number of cases informed**					**2539**	**53**

*Based on this discussion and on the results of the tables in the Supplementary Note, we conclude that the schemes with* $m=(3^k,\ldots ,3)$ *are also good because they produce some of the smallest overflows when running the strategy in parallel. Even when subdividing the pools in two would produce less overflow, these strategies require more stages and, consequently, more time to identify the infected samples than those in which the pools are subdivided in three. In clinical laboratories the turnaround time directly impacts on the productivity. Thus, the schemes with* $m=(3^k,\ldots ,3)$ *would be preferable.*

## ddPCR for increased sensitivity and verification purposes

All tests carry some level of uncertainty. Detecting viral genomes with pool testing has the additional problem of the dilution introduced by the pooling. The increased sensitivity of ddPCR, particularly to detect SARS-CoV-2 RNA^21,23,24,26,32^, is a great advantage. The fact that ddPCR quantifies the analyte at the end of the test without the need of calibration curves^33,34^ is another advantage. As shown here, this quantification can be used to increase the results reliability and reduce misclassification errors. In ddPCR the volume that goes in the reaction tube is subdivided into many (20,000) sub-volumes. If the fraction of sub-volumes with detected RNA is bounded away from 0 or 1, a range of possible values for the RNA concentration can be obtained. When testing a pool of $m_j$ samples, the estimated RNA concentration corresponds to (see Supplementary Note):

5 $$\begin{aligned} c_R^{(j)}= \frac{E{N}_{R}^{(j)}}{V_s}=\frac{1}{m_j}\sum _{i=1}^{m_j} \tilde{c}_{R;i}, \end{aligned}$$

where the indices, $i=1,\ldots ,m_j$, identify the samples contained in the pool; $\tilde{c}_{R;i}$ is the RNA concentration of the *i*-th sample extracted from the corresponding individual (what we call the *original sample* in the Supplementary Note) and ${E{N}_{R}^{(j)}}$ is the expected number of RNA molecules in a volume of the pool equal to the *sample volume*, $V_s$. $V_s$ does not involve any dilution with respect to the original sample exctracted from the individual. The preparation for the test can be accounted for as introducing a dilution of factor, *D*, that does not change the number of RNA molecules; i.e., ${E{N}_{R}^{(j)}}$ is also the expected number of RNA molecules in the test volume, $V_t=DV_s$, of the given pool. Under the usual assumptions for the quantification in ddPCR tests, the concentration will be estimated if it satisfies $500/{\mathrm{ml}} \lesssim c_R^{(j)}/D\lesssim 7.4 10^6/{\mathrm{ml}}$ (see Supplementary Note).

### Minimum viral load detectability and maximum pool size using ddPCR

In pooled testing, the worst case scenario for detectability purposes is that in which all the samples but one correspond to healthy individuals. Let us consider an $m_j$-sample pool in which the *i*-th sample corresponds to an infected individual whose viral genome concentration is $\tilde{c}_{R;i}$ (defined as in Eq. (5)). The probability of having at least one viral RNA molecule in the pool, $P(N_{R}^{(j)}\ge 1)$, as a function of $\tilde{c}_{R;i}$ is then (see Supplementary Note):

6 $$\begin{aligned} P(N_{R}^{(j)}\ge 1)= 1-\exp \left( -\frac{\tilde{c}_{R;i}V_t}{m_jD}\right) . \end{aligned}$$

**Figure 6:**
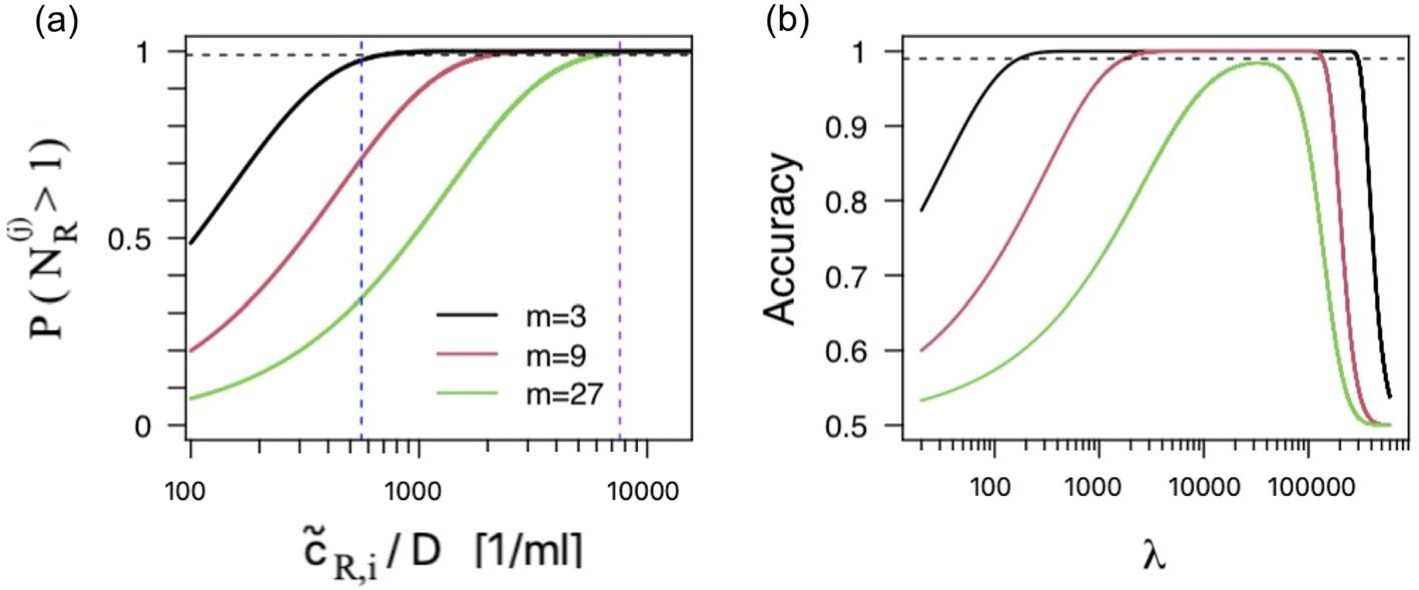
ddPCR and RNA quantification. (**a**) Probability of having at least one RNA molecule in the testing volume of an $m_j$ pool (black: $m_j=3$, red: $m_j=9$, green: $m_j=27$) in which only one sample corresponds to an infected individual whose viral genome concentration is $\tilde{c}_{R;i}$. The blue vertical line corresponds to the minimal viral RNA amount (quantified with ddPCR) detected in individual samples of people infected with SARS-CoV-2 and the one in purple to the average minus SEM (651-501 copies/test) detected in nasal swabs^35^). The dashed horizontal line corresponds to the probability $P=0.99$. (**b**) Accuracy of the test that discriminates between having one flawed sample in an *m*-sample pool and having none, as a function of the expected number of RNA molecules, $\lambda$, in a volume of size, $V_s$, of the original sample, when a ddPCR is run on the pool subdividing the testing volume, $V_t=DV_s$, into $M=20,000$ sub-volumes. The pool sizes illustrated are $m=3$ (black), $m=9$ (red) or $m=27$ (green). Here $\lambda$ is the expected number over the whole human population. The dotted line corresponds to the accuracy, 0.99.

We show in Fig. 6a the plot of $P(N_{R}^{(j)}\ge 1)$ as a function of $\tilde{c}_{R;i}/D$ for $V_t=20\mu l$ and various $m_j$. The horizontal line corresponds to $P=0.99$. The vertical lines correspond to the SARS-CoV-2 RNA content quantified by ddPCR in the test volume of individual samples reported in^35^: the curve in blue corresponds to the minimal load detected (11.1 copies/test) and the one in purple to the average minus SEM (651-501 copies/test) detected in samples of nasal swabs (the specimens with lowest viral content reported in^35^). There we observe that a single sample with the minimal viral load detected in^35^ could be detected, according to our calculation, in $>97\%$ of the cases in 3-sample pools and in $>70\%$ of the cases in 9-sample ones. The lowest end of the average obtained in nasal swabs could be detected with 3, 9 or 27-sample pools in over 99% of the cases. Considering that the average viral loads detected in throat swabs and sputum samples were, respectively, $\sim 4$ and 27 times the average in nasal swabs, it seems that a single infected sample would be detected in a fairly large fraction of cases using pools of 27 or more samples of these specimens. We analyze this aspect in more detail in the Supplementary file using the estimates of virion distribution among infected people reported in^36^.

#### ddPCR quantification and the identification of “flawed” pooled samples

PCR tests also detect reporters besides the desired analyte to confirm that the reaction has proceeded correctly as well as the integrity of the sample, avoiding false negative results due to inappropriate handling. For the detection of viral RNA in human samples, this confirmatory reporter is a piece of human RNA. If the control RNA is not detected, the result of the test is not reported and a new sample from the individual is requested. When multiple samples are pooled, the human RNA contained in only one of them can mask the absence of such RNA in the others. The only negative results that could be informed reliably would be those tested at the level of individual samples, i.e., a small fraction of those that could in principle be informed as negative (see e.g., Fig. 3). The pooling strategy could then be applied mainly to identify those that are detectably infected which could still serve for epidemiological purposes and for continuous validation of the method. As analyzed here, the quantification given by ddPCR can serve to enlarge the truly confirmed set of negative tests.

To analyze whether it is possible to detect the presence of *flawed* (with undetectable levels of the control human RNA) samples in pools using the quantification that ddPCR gives, we introduce the notation

7 $$\begin{aligned} \lambda _i \equiv V_{s} \tilde{c}_{R;i} = \frac{V_{t}}{D} \tilde{c}_{R;i}, \end{aligned}$$

to denote the *mean number* of human RNA molecules of the *i*-th individual in a volume of size, $V_s$. In an *m*-sample pool in which the *i*-th individual sample occupies a volume, $V_s/m$, the number of human RNA molecules it will contribute with has mean, $\lambda _i/m$. To consider the human to human variability, we assume that the values, $\lambda _i$, of the samples that are not flawed are (independent) instances of a random variable with a certain distribution at the level of the human population. Given the multiple factors that affect the human RNA content in the drawn samples, the most parsimonious hypothesis is to assume that this distribution is Normal of mean, $\lambda$, and standard deviation, $\sigma$ (see Supplementary Note). We then consider that an *m*-sample pool with $\ell$ flawed samples is characterized by a sequence, $\lambda _1, \lambda _2, \ldots \lambda _{m-\ell }$ of independent identically (Normal) distributed (i.i.d.) random variables and that $\lambda _{m-\ell +1}=\ldots =\lambda _{m}=0$. We now consider a ddPCR experiment in which the testing volume is divided into *M* sub-volumes. Proceeding as explained in the Supplementary Note, we compute the likelihood that the ddPCR does not detect any human RNA molecules in *x* of the *M* sub-volumes under the two following situations: (i) the *m* individual samples were “good”; (ii) only one of the *m* samples was “flawed”. Under certain assumptions, these two situations will be distinguished with accuracy (fraction of instances for which the classification is correct):

8 $$\begin{aligned} {\mathrm{Accuracy}} =&\Phi \left( \frac{x_c-Me^{-\lambda /M}}{\sqrt{M(1-e^{-\lambda /M})e^{-\lambda /M}}}\right), \quad x_c=M\left( 1-\left( 1-\frac{m M}{\lambda }\left( \log \left( 1-e^{-\lambda (1-1/m)/M}\right) \right. \right. \right. \nonumber \\&\left. \left. \left. - \log \left( 1-e^{-\lambda /M}\right) \right) \right) ^{-1}\right) \end{aligned}$$

where $\Phi (z)=\int _{-\infty }^z \frac{dx}{\sqrt{2 \pi }}\exp ({-\frac{x^2}{2}})$. For very small and very high $\lambda$ values Eq. (8) is not valid (see Supplementary Note). Nevertheless, for those values the accuracy tends to 1/2. We show in Fig. 6b a plot of the accuracy given by Eq. (8) as a function of $\lambda$, for 3 pool sizes ($m=3,9, 27$) and $M=20,000$, a standard for ddPCR tests. We observe in the figure that, for the accuracy to be greater than 0.99 (dotted line) $\lambda$ can vary within the interval, $[172, 2.96\, 10^5]$, if the pool size is $m=3$. In the $m=9$ case, it must satisfy $1.7\, 10^3\le \lambda \le 1.36\, 10^5$. For a pool with $m=27$ we cannot obtain a 0.99 accuracy for any value of $\lambda$. The general rule is that, if *m* is below a certain value, $m_{max}$, there is a finite range of $\lambda$ values over which the accuracy is larger than 0.99. This range gets narrower as *m* increases. If $m>m_{max}$ the accuracy is below 0.99 for all values of $\lambda$. The range of $\lambda$ values for which the discrimination test has high accuracy may be translated into RNA concentrations, $c_R$, as done in Eq. (7): $c_R=\lambda /V_s=D \lambda /V_t$. Using $V_t=20$ μl (a typical testing volume) we obtain that the concentrations in the orginal sample, $c_R$, must satisfy $8.6\, 10^3/{\mathrm{ml}}< c_R/D< 1.48\, 10^7/{\mathrm{ml}}$ for $m=3$ and $8.5\, 10^4/{\mathrm{ml}}< c_R/D < 6.8\, 10^6/{\mathrm{ml}}$ for $m=9$. The range in the $m=9$ case is fully contained within the range for which ddPCR can provide an accurate estimation of the RNA concentration (see Supplementary Note). The highest end for the $m=3$ case goes beyond the limit set by ddPCR; their overlap occurs for: $8.6\, 10^3/{\mathrm{ml}}< c_R/D<7.4\, 10^6/{\mathrm{ml}}$. Assessing whether these conditions can be satisfied in actual situations requires further studies. In case that this discrimination could be possible for two successive stages, a re-checking could be done as described in the following Section. The nestedness of the strategy could also be used for further validation.

#### Combining the nested strategy with ddPCR quantification for self-consistency tests and to estimate RNA concentration in samples with high viral load

We discuss now how the nested nature of the strategy can be used for test verification. Let us then consider an $m_j$-sample pool, $m_j>1$, that tests positive at the *j*-th stage. At stage $j+1$, the samples of this pool are grouped into $m_j/m_{j+1}$ pools with $m_{j+1}$ samples each. Let us use the subscript, $\ell$, $1\le \ell \le m_j/m_{j+1}$, to label these “sub-pools”. Given Eq. (5), the expected number of RNA molecules added over all these subpools is:

9 $$\begin{aligned} \sum _{\ell =1}^{m_{j}/m_{j+1}} E{N}_{R,\ell }^{(j+1)} =V_s \sum _{\ell =1}^{m_{j}/m_{j+1}}c_{R;\ell }^{(j+1)}= \sum _{\ell =1}^{m_{j}/m_{j+1}} \frac{V_s}{m_{j+1}}\sum _{i=(\ell -1)m_{j+1}+1}^{m_{j+1}} \tilde{c}_{R;i}= \frac{V_s}{m_{j+1}}\sum _{i=1}^{m_{j}} \tilde{c}_{R;i} =V_s \frac{m_j}{m_{j+1}} c_R^{(j)} , \end{aligned}$$

where $c_{R;\ell }^{(j+1)}$ is the RNA concentration, before dilution, that is estimated for the $\ell$-th sub-pool at the end of the ddPCR test of the $j+1$-th stage (see Supplementary Note for more details). Eq. (9) imposes a condition that the RNA concentrations derived from the ddPCR tests performed on the same subset of samples at two subsequent stages should satisfy. The ddPCR quantification actually gives an interval of possible concentration values whose width can be relatively small so that condition (9) can be verified quite accurately (Supplementary Note). The advantage of the nested strategy is that the set of samples that would require some re-testing, if an inconsistency is found, is reduced with respect to strategies that are either non-adaptive or that mix up the samples at random at every stage. The nested nature of the strategy also allows the identification of the pools that the infected samples belonged to at any given stage. This is of help to quantify the viral genome content in samples with high loads: if at the last stage the viral genome concentration exceeds the upper bound allowed for a reliable quantification with ddPCR, then, in certain cases it will be possible to deduce it from the concentration determined at a previous stage. Let us consider the example of Fig. 1 and use the same labeling for viral RNA concentrations that we use for pools and samples. Let us then assume that $\tilde{c}_{020}$ is so large that $\tilde{c}_{R;020}/D$ exceeds the upper bound ($\sim {7.4\, 10^6}ml^{-1}$) for a reliable quantification with ddPCR (see Supplementary Note). Knowing the genealogy of $W_{020}$ allows us to infer that this is the only infected sample in the second-stage pool, $W_{02}$ and in the first-stage one, $W_{0}$. Applying Eq. (5) to these two pools we obtain:

10 $$\begin{aligned} c_{R;0}^{(1)}= \frac{1}{m_1}\sum _{i_2=0}^{2}\sum _{i_3=0}^{2} \tilde{c}_{R;0i_2i_3}=\frac{1}{m_1}\tilde{c}_{R;020};\quad c_{R;02}^{(2)} = \frac{1}{m_2}\sum _{i_3=0}^{2} \tilde{c}_{R;02i_3}=\frac{1}{m_2}\tilde{c}_{R;020}. \end{aligned}$$

It is possible that $c_{R;0}^{(1)}$ and/or $c_{R;02}^{(2)}$ could be quantified giving an estimate of $\tilde{c}_{R;020}$ through one of Eqs. (10).

## Conclusion

We have studied in detail the nested test pooling strategy of^10^ and optimized in^15^, providing a clear guidance to establish the number of samples in each pool and how to partition the positive pools for the identification of infected individuals in the presence of different constraints. Assuming that ddPCR is used to detect the presence of the RNA piece of interest, we have determined the limit of viral genome detection in pools, particularly in the case of SARS-CoV-2, and establish the range of human RNA content per sample useful as an indicator of the presence of unidentifiable samples in a pool. We have also shown how the combination of the ddPCR quantifying capabilities with the nested nature of the strategy can be used in different ways for verification purposes. The simplicity of the strategy, its easily parallelization and the advantages of its nested nature make it a very valuable option to faithfully detect viral genomes in a cost effective manner.

## Methods

### Analytic formulas

The expected number of tests per initial pool at each stage *j* of the hierarchical strategy^10^ with $k+1$ stages is (see Supplementary Note):

11 $$\begin{aligned} E{T}^{(j)}_{k}(m,p)&=\frac{m_1}{m_j} \bigl (1-(1-p)^{m_{j-1}}\bigr ),\qquad 2\le j \le k+1 , \end{aligned}$$

the expected total number is $ET_k=1+\sum _{j=2}^{k+1} E{T}^{(j)}_{k}$, and the cost of the strategy is:

12 $$\begin{aligned} D_{k}(m,p)&\equiv \frac{ET_k}{m_1}=\frac{1}{m_1}\left( 1 + \sum _{j=2}^{k+1}E{T}^{(j)}_{k}\right) =\frac{1}{m_1} + \sum _{j=2}^{k+1} \frac{1}{m}_j \bigl (1-q^{m_{j-1}}\bigr ), \end{aligned}$$

where $q=1-p$ is the probability that an individual is not infected. The cost of the strategy in the presence of classification errors is presented in the Supplementary Note.

Restricting the analyses to strategies with $m=(\mu ^k,\ldots ,\mu )$, $\mu >1$, we proved^15^ that the optimal one has $k=k_\mu (p)$ with:

13 $$\begin{aligned} k_\mu (p) \equiv \Big \lfloor \log _{\mu } \Bigl (\frac{1}{\log _{\mu }( 1/(1-p) )}\Bigr )\Big \rfloor =\Big \lfloor \log \Bigl (\frac{\log (\mu )}{\log ( 1/(1-p) )}\Bigr )/\log (\mu )\Big \rfloor , \end{aligned}$$

where $\lfloor \zeta \rfloor$ is the largest integer, $n_\zeta$, such that $n_\zeta \le \zeta$. We further proved^15^ that the strategy with $m=(3^{k_3(p)},\ldots ,3)$ and $k_3(p)$ given by Eq. (13), $\mu =3$, is the optimal nested strategy for almost all values of *p* and its cost is of the same order of magnitude, $O\big (p\log (1/p)\big )$, as that of the optimum for the other values. For the strategies with $m=(\mu ^k,\ldots ,\mu )$, the standard deviation of the number of tests per individual is given by (see Supplementary Note):

14 $$\begin{aligned} \sigma (m,p)\frac{1}{\mu ^{k-1}} \left( \sum _{i=1}^{k} \mu ^{i-1}\bigl (1-q^{m_i}\bigr )\left( q^{m_i}+2\sum _{j=1}^{i-1}q^{m_j}\right) \right) ^{1/2} . \end{aligned}$$

### Nested pooling method: stochastic numerical simulations and sample labeling

The stochastic simulations are characterized by the infection probability, *p*, the number of stages before the individual testing, *k*, the sequence of pool sizes, $(m_1,m_2,\ldots ,m_k)$, and the number of realizations (or of initial pools, $N_w$). A sequence of $m_1 N_w$ (pseudo) random numbers is generated with uniform probability over [0, 1) and the infected samples are assigned to the positions where the random number is $\le p$. The samples in each initial pool are chosen sequentially along the $m_1N_w$-long sequence. The strategy is applied as described in Fig. 1 choosing the samples in the testable pools of each stage sequentially along the initial sequence as well. Keeping the same ordering, at every stage, of all the initial individual samples, we can associate $(k+1)$ subscripts, $i_1 i_2 ... i_{k+1}$, to each of them that serve to uniquely identify the pools they belong to at any given stage. This is apparent in Fig. 1 where the individual samples are represented along a horizontal line with the first one starting from the left being labeled by the subscripts 000 and the last one by 022. In this case we are considering only one initial pool, $W_0$, and that is why the first subscript is $i_1=0$. Some of the histograms obtained with the simulations also include theoretical computations. These are done counting exhaustively the combinations that lead to the various end results in terms of the number of tests, for numbers that do not exceed a certain value (see the Supplementary Note).

## Supplementary Information

Supplementary Information.

## Data Availability

Accession codes: All the code written is available at http://www.pooling.df.uba.ar.
