# Supplementary material for: Nested pool testing strategy for the diagnosis of infectious diseases
Source: Sci Rep. 2021 Sep 13;11:18108. doi: 10.1038/s41598-021-97534-7 (PMC8438083; doi:10.1038/s41598-021-97534-7)
Supplement: Supplementary file 1 — Supplementary Information. [file 41598_2021_97534_MOESM1_ESM.pdf]

Supplementary Note for:
"Nested pool testing strategy for the diagnosis of infectious diseases"

Inés Armendáriz[1], Pablo A. Ferrari[1], Daniel Fraiman[2], José M. Martínez[3], Hugo G. Menzella[4], Silvina Ponce Dawson[5*],

1 Departamento de Matemática & IMAS, FCEN-UBA & CONICET, Ciudad de Buenos Aires, Argentina
2 Departamento de Matemática y Ciencias, UDESA & CONICET, Victoria, Provincia de Buenos Aires, Argentina
3 Department of Applied Mathematics, Institute of Mathematics, Statistics, and Scientific Computing (IMECC), University of Campinas, Campinas SP, Brazil
4 Departamento de Tecnología & IPROByQ, FBIOyF-UNR & CONICET, Rosario, Provincia de Santa Fe, Argentina
5 Departamento de Física & IFIBA, FCEN-UBA & CONICET, Ciudad de Buenos Aires, Argentina
* silvina@df.uba.ar

1 Background on pool testing

Group or pool testing has been extensively studied for two main applications: (*i*) to identify "defective" units (in the present case, infected individuals) when the prevalence, p, defined as the probability of being defective, is relatively low [1]; (*ii*) to estimate p [2]. In the present manuscript we mostly focus on the identification problem. This is the key aspect for the expansion of the testing capacity; accepting that a negative result in a single reaction indicates that all the individuals whose samples are included in the tested pool are not infected.

The first proposal of a pooling strategy for this aim was made by Dorfman [3] in 1943. In this strategy, pools are formed containing a number, m, of individual samples. One test is run on each pool to detect the presence of a defective or infected sample in it. Assuming that the detection is sensitive enough, the samples in the pools that test negative in this first stage are identified as non-infected. All the samples that belong to pools that test positive are tested individually at a second stage. The identification of the samples that belong to pools that test negative at first is done with only one test. The identification of those that belong to pools that test positive at first requires $m + 1$ tests instead. A large reduction in the number of tests will then be achieved depending on the relative fraction of pools that test positive at the first stage. This scheme is an example of a class of models that are called adaptive or hierarchical or sequential. In this type of models the actions to be taken at any given stage depend on the test results of the previous stages. The two-stage strategy of Dorfman's was subsequently improved by others [4–8] including more stages to further reduce the number of tests that are necessary to identify the infected samples. In particular, the method of [6], which was recently optimized in [9], achieves an important reduction at the expense of having a random, in principle large, number of stages. Alternatively, non-adaptive methods can be applied, in which the way in which the samples are pooled and tested is established before having the result of any test. This definition of the pools *a priori* allows an easy

parallelization of the strategy [10] and, depending on the method, can also help to reduce the impact of test errors by including all individual samples in more than one pool. One of the most common such strategies is array testing in which each sample is included in at least two pools and if both pools test negative then the individual samples are labeled as not infected, otherwise they are tested individually [11–13]. There are also mixed strategies, called semi-adaptive, that try to exploit the advantages of the different types of approaches simultaneously [14]. According to [10], the mixed strategy of [14] is the best two stage strategy in terms of reducing the number of tests per individual.

Various pool testing strategies have been analyzed specifically for the case of SARS-CoV-2. In [15] Dorfman's algorithm is applied including the use of replicates to check for false negatives or positives. In [16], Dorfman's strategy is compared numerically with adaptive and non-adaptive methods that use, at some stage, binary splitting. The work in [17] evaluates numerically the performance of two-dimensional array pooling in which the individual samples are organized in an $r \times c$ array. In [18] a D-dimensional array pooling strategy is evaluated assuming that there are very few infected samples among the tested ones. This strategy is being used for large scale testing in Rwanda [19]. In [20], both Dorfman's and array algorithms are evaluated on practical implementations. In [21] a non-adaptive group testing approach is presented which uses a combinatorial pooling strategy that is based on compressed sensing and is designed to maximize the identification of the positive samples. So far, sample pooling strategies for SARS-CoV-2 nucleic acid detection have mostly been explored for the current gold standard test, RT-qPCR [22], finding that, for large enough viral loads, the identification of a single infected individual is possible in pools of up to 100 samples (1 infected + 99 negative samples). Despite the initial excitement, it is accepted that in RT-qPCR the target can go unidentified if present in small amounts, and that samples frequently contain inhibitors of the PCR reaction [23]. Moreover, diagnostic sensitivity in symptomatic patients was reported to be in the range of 60%–90% [24]. Thus, for samples with low viral loads, the pooling associated dilution will further reduce the detection of infected individuals to unacceptable levels. The risk of increasing the number of false negative results has raised concerns and delayed the approval of group testing protocols, limiting pool sizes to single digit numbers. This has reduced the impact of pooling strategies and, consequently, the expansion of the world testing capacity.

2 Nested pooling strategy

The hierarchical pooling strategy analyzed in the paper is characterized by a number of stages, $k+1$, and a sequence of pool sizes, $m = (m_1, \ldots, m_k)$, where $m_1 > \cdots > m_k > 1$ and m_j is a multiple of m_{j+1} for all j. At the last $(k+1)$ stage the samples are tested individually, *i.e.*, $m_{k+1} = 1$. It was first introduced for $k = 2$ [7] and later generalized to any number of stages [8]. Given the infection probability, p, the optimal strategy is characterized by values, k and m, that minimize the cost (the expected number of tests per individual). In [25] we derived analytic expressions for the parameters that define the optimal strategy for any given p. We present in what follows a brief description of the strategy and the main results obtained in [25] that are used in the present paper. Readers interested in our approach can try it at [26]. Given that different facilities might have different constraints, the site offers the possibility of choosing one or various constraints to start with and gives not only the optimal strategy given the constraints but several suboptimal ones as well. In this way users can choose the strategy that best meets their needs, even if it is not the absolute optimal in terms of resource savings.

2.1 Presentation of the strategy

Let us assume that N individuals have to be tested and that p is the probability that anyone of them is infected. At the first stage we subdivide the N samples into disjoint groups of size, m_1, and combine the m_1 samples of each group into a single pool that is tested. Once the first-stage tests are performed, we subdivide the samples of each of the pools that detected the presence of at least one infected sample (*i.e.*, that tested positive) into m_1/m_2 pools of m_2 samples each. We perform the test on these m_2 sample pools and proceed as before: those that test positive are subdivided in m_2/m_3 pools of m_3 samples each. The procedure is iterated until the $(k+1)$-th stage is reached at which all the individual samples of the pools that tested positive at the $k-$th stage are tested.

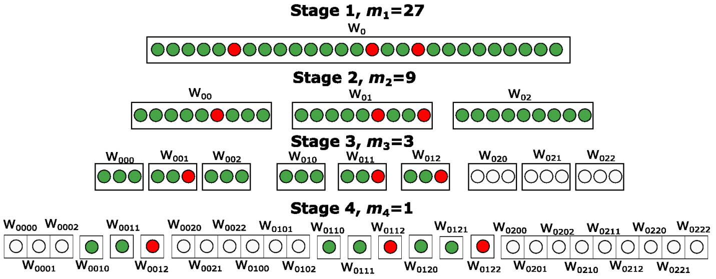

Fig 1. Schematic depiction of the nested pool strategy with $k = 3$ and $m = (27, 9, 3)$ applied to an initial pool (rectangle) with samples (circles) coming from the 27 individuals in the set, W_{i_1} with $i_1 = 0$, three of which are infected (red circles in the figure). Each pool of each stage is subdivided into 3 pools at the following stage. The figure illustrates the labels that we assign to the different pools at the various stages and how this can be done *a priori*. In any practical implementation of the strategy, only the pools with infected samples pass to the following stage. In the example of the figure, the test performed on W_0 is positive (because there are samples of infected individuals in W_0). The subpools, W_{00}, W_{01} and W_{02}, of W_0 are then tested at the second stage, the first two of which turn out to be positive because they contain samples from infected individuals. All the sub-pools of W_{00}, W_{01} and W_{02}, are depicted in the third-stage row of the figure, but only those that are tested at this stage contain circles that are colored in red or green if infected or not, respectively. The grey circles correspond to samples identified as not infected at previous stages. Thus, only W_{000}, W_{001}, W_{002}, W_{010}, W_{011} and W_{012} are tested at the third stage, three of which turn out to be positive so that 9 samples are tested individually at the last (4th) stage. The total number of tests that are performed in this case is 19, which is smaller than 27, and the cost is 0.7. As illustrated in Fig. 1 of the paper, this situation occurs in less than 5% of the cases for which the strategy with $k = 3$ and $m = (27, 9, 3)$ is optimal.

In order to understand the optimization of the procedure it is best to introduce a labeling of the pools at all stages from the very beginning, as if none of them ever tested negative. To this end, let us call, W_{i_1}, the set of individuals whose samples are contained in the i_1-th pool of the first stage. We subdivide this set into m_1/m_2 subsets with m_2 individuals each. Let us call $W_{i_1 i_2}$ the sets of individuals whose samples, at the second stage, are in the i_2-th subpool of the first stage i_1-th pool. We repeat this labeling at all stages, so that at the $k-$th one we have subsets $W_{i_1 \ldots i_k}$. For the sake of simplicity, we will also refer to the pools of samples with these same names, $W_{i_1 \ldots i_k}$. To each of these subsets we assign a variable, ϕ, such that $\phi = 0$ if there is at least one infected individual in the subset and $\phi = 1$ otherwise. We introduce the notation $\phi_{i_1} = \phi(W_{i_1})$, $\phi_{i_1 i_2} = \phi(W_{i_1 i_2})$, $\ldots$, $\phi_{i_1 \ldots i_k} = \phi(W_{i_1 \ldots i_k})$. We illustrate this labeling in Fig. 1 where we depict one first stage pool, W_0, and its subdivision for an example with 4 stages ($k = 3$) that starts with $m_1 = 27$ and such that each (positive) pool is subdivided into three new pools at each successive stage. The name "nested" reflects the fact that samples that come from different positive pools at a given stage are not mixed up for testing at subsequent stages.

2.2 Number of tests

Using the labeling illustrated in Fig. 1 and the definition of ϕ we can write the number of tests, $T_k^{(j)}(m,p)$, that are needed to perform at each stage, $2 \le j \le k+1$, of the $(k+1)$-stage strategy with pool sizes, $m=(m_1,...,m_k)$, to identify the infected individuals in each first-stage set, W_{i_1}. This number is equal to m_{j-1}/m_j times the number of pools that test positive at the $j-1$ stage:

$$T_k^{(j)}(m,p) = \frac{m_{j-1}}{m_j} \sum_{i_2=0}^{\frac{m_1}{m_2}-1} \sum_{i_3=0}^{\frac{m_2}{m_3}-1} \cdots \sum_{i_{j-1}=0}^{\frac{m_{j-2}}{m_{j-1}}-1} \left(1-\phi_{i_1 i_2 \ldots i_{j-1}}\right), \qquad 2 \le j \le k+1, \tag{1}$$

where we have introduced $m_{k+1}=1$. The only non-zero terms in Eq. (1) correspond to the m_{j-1}-sample pools with $\phi_{i_1 i_2 \ldots i_{j-1}} = 0$ (*i.e.*, with infected samples). The probability that $\phi_{i_1 i_2 \ldots i_{j-1}} = 0$ is $(1-(1-p)^{m_{j-1}})$ so that the expected value of $T_k^{(j)}(m,p)$ is:

$$ET_k^{(j)}(m,p) = \frac{m_1}{m_j}\left(1-(1-p)^{m_{j-1}}\right), \qquad 2 \le j \le k+1. \tag{2}$$

Taking into account that all pools are tested at the first stage, the expected value of the total number of tests that is needed to identify the infected individuals in any first-stage pool with the $m=(m_1,...,m_k)$ strategy is then given by:

$$ET_k = 1 + \sum_{j=2}^{k} \frac{m_1}{m_j}\left(1-(1-p)^{m_{j-1}}\right) + m_1\left(1-(1-p)^{m_k}\right). \tag{3}$$

In the case $k=1$ this is just the formula computed by Dorfman [3]. The computation for all k seems to appear first in Kotz and Johnson [7], see also the monograph by Johnson, Kotz and Wu [8]. More recently the formula appears in [11–13, 27]. This last paper generalizes the formula to the case of individual dependent prevalences and variable pool-sizes. All these papers study the role of sensitivity and accuracy.

Certain approximations hold when the number of samples that correspond to infected individuals is low enough so that there is at most one infected sample in each pool. In such a case, at any given stage, only one pool "makes it" to the following stage. Thus, the total number of tests that have to be performed for each of these pools is:

$$\hat{T}_k = 1 + m_k + \sum_{j=2}^{k} \frac{m_{j-1}}{m_j} = 1 + k\mu, \tag{4}$$

and the number of tests per individual can be written as:

$$\overline{D}_k(m_1,\ldots,m_k,\tilde{p}) = \frac{1}{m_1} + m_k\tilde{p} + \tilde{p}\sum_{j=2}^{k}\frac{m_{j-1}}{m_j}, \tag{5}$$

with $\tilde{p}=1/m_1$. The latter is equal to the expected number of tests per individual for realizations with at most one infected sample per initial pool if we set $\tilde{p}$ equal to the infection prevalence, $\overline{p}$. It also coincides with the linearization of Eq. (21) replacing, $\tilde{p}$, by the infection probability, p. We proved [25] that the cost of the optimal nested strategy is smaller than the one given by Eq. (22). This is reflected in the example of Fig. 3 (a). Eqs. (4) and (22) with $\tilde{p}=1/m_1$ also hold when there is more than one infected sample in the initial pool that remain in the same pool up to the k-the stage.

The variance of the number of tests can also be computed analytically [25]. We only quote here the result for the special case of the strategies in which the ratio of

consecutive pool sizes is constant and equal to m_k. As described later, a strategy of this type (with $m_k = 3$) is the optimal for most values of the infection probability, p. The variance for the strategies with $m = (\mu^k, \mu^{k-1}, \dots, \mu)$, $\mu > 1$, can be written as [25]:

$$\begin{aligned} \mathrm{Var}\big(T_k\big) &= \mu^2 \Big\{ \sum_{i=1}^{k} \mu^{i-1} \big(1 - q^{m_i}\big) \Big[q^{m_i} + 2 \sum_{j=1}^{i-1} q^{m_j} \Big] \Big\} && (6) \\ &= \mu^2 \Big\{ \sum_{i=1}^{k} \mu^{i-1} \Big(1 - q^{\mu^{k-i+1}}\Big) \Big[q^{\mu^{k-i+1}} + 2 \sum_{j=1}^{i-1} q^{\mu^{k-j+1}} \Big] \Big\}, && (7) \end{aligned}$$

where $q = 1 - p$. This variance gives a standard deviation for the number of tests per individual:

$$\sigma(m,p) = \frac{1}{\mu^k} \sqrt{\mathrm{Var}\big(T_k(m,p)\big)} = \frac{1}{\mu^{k-1}} \left(\sum_{i=1}^{k} \mu^{i-1} \big(1 - q^{m_i}\big) \left(q^{m_i} + 2 \sum_{j=1}^{i-1} q^{m_j} \right) \right)^{1/2}. \tag{8}$$

Given a nested strategy, the probability of having to perform a certain number of tests can also be computed analytically as a function of the infection probability, p, using combinatorics. The problem becomes increasingly complicated as the size of the initial pool gets larger and the number of possible outcomes also increases. We hereby describe how to proceed in the case of the strategy illustrated in Fig. 1, which has $k = 3$ and $m = (27, 9, 3)$. Given an initial pool, the total number tests, T, that will have to be performed under such strategy can take on ten possible values: $\{1, 7 + 3 \times 1, 7 + 3 \times 2, \dots, 7 + 3 \times 9\}$. The procedure consists in counting in how many ways one can end up performing a number of tests, T, equal to one of these ten values, given a number of infected samples that can go from 0 through 27, pondering the various cases by their probability of occurrence. We limit the description to computing the probabilities of occurrence of the first few possibilities, but the procedure can be extended to all. Let us then call *minimal subpools* the sample pools associated to the nine sets, $W_{i_1 i_2 i_3}$, $0 \le i_1, i_2, i_3 \le 2$ (third row in the scheme of Fig. 1) and *maximal subpools* those associated to the three sets $W_{i_1 i_2}$, $0 \le i_1 i_2 \le 2$ (second row in Fig. 1).

The first case, $T = 1$, occurs when there are no infected samples in the initial pool. This occurs with probability:

$$P(T = 1) = (1 - p)^{27}. \tag{9}$$

The second case, $T = 10$, occurs when all the infected samples of the pool belong to the same minimal subpool. A minimal subpool can have 1, 2 or 3 infected samples. To compute the probability we first fix the number of infected samples and then count in how many ways we can distribute them so that they all belong to the same minimal subpool. Finally we add over the situations with 1, 2 or 3 infected samples with their corresponding probability of occurrence. We obtain:

$$P(T = 10) = \sum_{\ell=1}^{3} \binom{3}{1} \binom{3}{1} G_\ell^a p^\ell (1 - p)^{27-\ell} \tag{10}$$

In this equation, the term, $\binom{3}{1}\binom{3}{1}$, corresponds to the number of ways in which a minimal subpool can be selected. The term, $G_\ell^a := \binom{3}{\ell}$, is the number of different configurations with ℓ infected samples in the minimal subpool selected. The factor, $p^\ell (1 - p)^{27-\ell}$, is the probability of having ℓ infected samples. The third case, $T = 13$,

occurs if all the infected samples are in exactly two minimal subpools that belong to the same maximal subpool. In this case,

$$P(T=13)=\sum_{\ell=2}^{6}\binom{3}{1}\binom{3}{2}G^b_\ell p^\ell(1-p)^{27-\ell}. \tag{11}$$

The term, $\binom{3}{1}\binom{3}{2}$, represents the number of ways in which two minimal subpools that belong to the same maximal subpool can be selected. The term, $G^b_\ell := \binom{6}{\ell} - 2\binom{3}{\ell}$, is the number of different configurations with ℓ positve infected samples in the subpool selected, considering $\binom{n}{\ell} = 0$ if $k > \ell$. T takes on the value 16 if all the infected samples are either in exactly three minimal subpools that belong to the same maximal subpool, or they are in exactly two minimal subpools that belong to two different maximal subpools. In this case:

$$P(T=16)=\sum_{\ell=2}^{6}\binom{3}{2}\binom{3}{1}\binom{3}{1}G^b_\ell p^\ell(1-p)^{27-\ell}+\sum_{\ell=3}^{9}\binom{3}{1}\binom{3}{3}G^c_\ell p^\ell(1-p)^{27-\ell}, \tag{12}$$

with $G^c_\ell = \binom{9}{\ell} - (\binom{3}{2}\binom{6}{\ell} - 3\binom{3}{\ell})$. Eqs (9)–Eqs (12) were used to compute the analytic results depicted in Fig. 2 of the paper.

The probability of the number of infected samples in a pool of the first ($N_i^{(1)}$), second ($N_i^{(2)}$) or third stages ($N_i^{(3)}$) can be computed similarly. In particular, for a first stage pool (with $m_1 = 27$) it is:

$$P(N_i^{(1)}=\ell)=\binom{27}{\ell}p^\ell(1-p)^{27-\ell},\quad \ell=\{0,1,\dots,27\}. \tag{13}$$

The computation for $N_i^{(2)}$ and $N_i^{(3)}$ is cumbersome. In order to simplify it, we compute the probabilities subject to the condition $0 < N_i^{(1)} \leq 4$ (instead of requesting only that $0 < N_i^{(1)}$) which is reasonable to compare it with the results of Fig. 3 of the paper. The computation is straightforward. We obtain:

$$\begin{aligned}P(N_i^{(2)}=0|0<N_i^{(1)}\leq 4)&=\frac{1}{P(0<N_i^{(1)}\leq 4)}\times\Big[\frac{2}{3}\binom{27}{1}p^1(1-p)^{26}+\\ &\left(\frac{2}{3}\binom{3}{1}\binom{9}{2}+\frac{1}{3}\binom{3}{2}\binom{9}{1}\binom{9}{1}\right)p^2(1-p)^{25}+\left(\frac{2}{3}\binom{3}{1}\binom{9}{3}+\frac{1}{3}2\binom{3}{2}\binom{9}{2}\binom{9}{1}\right)p^3(1-p)^{24}\\ &+\left(\frac{2}{3}\binom{3}{1}\binom{9}{4}+\frac{1}{3}\binom{3}{2}\left(2\binom{9}{1}\binom{9}{3}+\binom{9}{2}\binom{9}{2}\right)\right)p^4(1-p)^{23}\Big],\end{aligned} \tag{14}$$

$$\begin{aligned}P(N_i^{(2)}=1|0<N_i^{(1)}\leq 4)&=\frac{1}{P(0<N_i^{(1)}\leq 4)}\times\Big[\frac{1}{3}\binom{27}{1}p^1(1-p)^{26}+\\ &\frac{2}{3}\binom{3}{2}\binom{9}{1}\binom{9}{1}p^2(1-p)^{25}+\left(\binom{3}{1}\binom{3}{1}\binom{3}{1}+\frac{1}{3}2\binom{3}{2}\binom{9}{2}\binom{9}{1}\right)p^3(1-p)^{24}\\ &+\left(\frac{1}{3}\binom{3}{2}\left(2\binom{9}{1}\binom{9}{3}+\frac{2}{3}\binom{3}{1}\binom{9}{2}\binom{9}{1}\binom{9}{1}\right)\right)p^4(1-p)^{23}\Big],\end{aligned} \tag{15}$$

$$\begin{aligned}P(N_i^{(2)}=2|0<N_i^{(1)}\leq 4)&=\frac{1}{P(0<N_i^{(1)}\leq 4)}\times\Big[\frac{1}{3}\binom{3}{1}\binom{9}{2}p^2(1-p)^{25}+\\ &\frac{1}{3}\binom{3}{1}\binom{9}{2}\binom{9}{1}p^3(1-p)^{24}+\left(\frac{1}{3}\binom{3}{1}\binom{9}{2}\binom{9}{1}\binom{9}{1}+\frac{2}{3}\binom{3}{2}\binom{9}{2}\binom{9}{2}\right)p^4(1-p)^{23}\Big]\end{aligned} \tag{16}$$

$$P(N_i^{(3)}=0|0<N_i^{(1)}\leq 4)=\frac{1}{P(0<N_i^{(1)}\leq 4)}\times[\frac{2}{3}\binom{27}{1}p(1-p)^{26}+$$
$$\left(\frac{2}{3}\binom{3}{1}\binom{3}{2}+\frac{2}{3}\binom{3}{2}\binom{9}{1}\binom{9}{1}+\frac{1}{3}\binom{3}{2}\binom{3}{1}\binom{3}{1}\right)p^2(1-p)^{25}+$$
$$\left(\frac{2}{3}\binom{9}{1}\binom{9}{1}\binom{9}{1}+\frac{1}{2}2\binom{9}{2}\binom{3}{2}\binom{3}{1}\right)p^3(1-p)^{24}+$$
$$\left(\frac{1}{3}\binom{9}{2}\binom{3}{2}\binom{3}{2}+\frac{2}{3}\binom{9}{4}\binom{3}{1}\binom{3}{1}\binom{3}{1}\binom{3}{1}\right)p^4(1-p)^{23}] \tag{17}$$

$$P(N_i^{(3)}=1|0<N_i^{(1)}\leq 4)=\frac{1}{P(0<N_i^{(1)}\leq 4)}\times[\frac{1}{3}\binom{27}{1}p(1-p)^{26}+$$
$$\frac{2}{9}\binom{9}{2}\binom{3}{1}\binom{3}{1}p^2(1-p)^{25}+(\frac{1}{9}2\binom{9}{2}\binom{3}{2}\binom{3}{1}+\frac{3}{9}\binom{9}{3}\binom{3}{1}\binom{3}{1}\binom{3}{1})p^3(1-p)^{24}+$$
$$\frac{1}{9}\binom{9}{2}2\binom{3}{3}\binom{3}{1})p^4(1-p)^{23}] \tag{18}$$

$$P(N_i^{(3)}=2|0<N_i^{(1)}\leq 4)=\frac{1}{P(0<N_i^{(1)}\leq 4)}\times[\frac{1}{3}\binom{3}{1}\binom{3}{1}\binom{3}{2})p^2(1-p)^{25}+$$
$$(\frac{1}{3}2\binom{3}{1}\binom{3}{1}\binom{3}{2}\binom{3}{1}+\frac{1}{6}\binom{3}{2}\binom{3}{1}\binom{3}{1}\binom{3}{1}\binom{3}{2})p^3(1-p)^{24}+$$
$$(\frac{1}{3}2\binom{3}{1}\binom{3}{2}\binom{3}{3}\binom{3}{1}+\frac{2}{6}\binom{3}{2}\binom{3}{1}\binom{3}{1}\binom{3}{2}\binom{3}{2}+\frac{1}{9}\binom{3}{3}\binom{3}{1}\binom{3}{2}\binom{9}{1}\binom{9}{1})p^4(1-p)^{23}] \tag{19}$$

We illustrate these computations in Fig. 3 and 4 (shown in green) where they are compared with the results of stochastic numerical simulations of the nested strategy performed over various initial pools as explained later.

2.3 Cost and optimization

The aim of pooling is to reduce the number of tests as much as possible. Let the *cost* be the expected number of tests per individual [8]:

$$D_k(m,p)\equiv\frac{1}{m_1}ET_k, \tag{20}$$

which, given Eq. (3), is equal to:

$$D_k(m,p)=\frac{1}{m_1}+\sum_{j=2}^{k}\frac{1}{m_j}\left(1-(1-p)^{m_{j-1}}\right)+1-(1-p)^{m_k}. \tag{21}$$

Some properties are derived from the linearization in p of Eq. (21) which is given by:

$$\overline{D}_k(m_1,\ldots,m_k,p)=\frac{1}{m_1}+m_kp+p\sum_{j=2}^{k}\frac{m_{j-1}}{m_j}. \tag{22}$$

This expression is similar to the expected number of tests per individual for realizations with at most one infected sample per initial pool if we replace p by the infection prevalence, $\overline{p}$. We proved [25] that:

$$D_k(m_1,\ldots,m_k,p)\leq\overline{D}_k(m_1,\ldots,m_k,p). \tag{23}$$

This implies that we can always expect our proposed strategy to produce, on average, a larger reduction in the number of tests per individual than the one predicted by Eq. (22).

The optimization problem consists in finding the value, k, and the sequence of pool sizes, m, that minimize (21) for a given p. We proved in [25] that, for $p \geq 1 - 3^{-1/3}$ it is best not to pool and that, for $p \in (2^{-51}, 1 - 3^{-1/3})$, the optimal nested strategy is of the form $(3^k, \ldots, 3)$ for most values of p interspersed with small p intervals over which it is $(3^{k-1}4, 3^{k-1}, \ldots, 3)$. In [25] we gave a precise description of the optimal values of k as functions of p and of the p-intervals over which one or the other scheme is best. We show the cost of the optimal strategy as a function of p in Fig. 2 (a). We conjecture it holds true for all $p \in (0, 1)$.

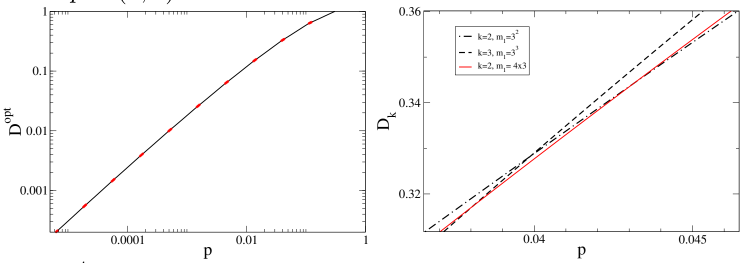

Fig 2. (a) Cost, D^{opt}, of the optimal nested strategy as a function of p in log-log scale. Red regions correspond to strategies with $m_1 = 4 \times 3^{k-1}$, the rest to $m_1 = 3^k$. We observe that, for every order of magnitude in p, the most likely best nested strategy is the one with $m = (3^k, ..., 3)$. (b) Cost of the strategies with $m = (3^2, 3)$ (black dashed-dotted curve), $m = (4 \cdot 3, 3)$ (red solid curve) and $m = (3^3, 3^2, 3)$ (black dashed curve). We observe that for the largest (smallest) probabilities displayed in the figure, the first (third) one is best. In between there is a small interval over which the best is the one with $m_1 = 4 \cdot 3$.

The costs of the strategies with $m = (3^3, 3^2, 3)$ (black dashed curve), $m = (4 \cdot 3, 3)$ (red solid curve) and $m = (3^2, 3)$ (black dashed-dotted curve) are shown in Fig. 2 (b). These strategies are optimal for $0.0146 \lesssim p \lesssim 0.0380$; $0.0380 \lesssim p \lesssim 0.0431$ and $0.0431 \lesssim p \lesssim 0.1098$, respectively [25]. Not only the interval of optimality for the strategy with $m_1 = 4 \cdot 3$ is very small compared to those of the other two (Fig. 2 (a)), but also the difference between the three costs is very small ($< 0.4\%$) over this interval. A similar behavior is observed for all k.

The paper of Black *et al* [27] also looked for the optimal within the family of nested strategies considered here. To this end, the cost of each possible strategy was computed and then minimized by inspection. The example of a 5-stage strategy with initial pool size, $m_1 = 18$, was worked out in detail. In [27] the optimal strategy is called ORC. Our optimal strategy corresponds to the ORC formula of [27] in the restricted case of no errors, equally sized groups and known homogeneous prevalence, p. Our theoretical contribution is an analytic expression for the optimal (unrestricted) strategy for any value of p. Since we have no formula for situations with further constraints, such as maximal group size and/or maximal number of stages, we have implemented a fast program (available at [26]) to find the 10 best strategies satisfying those restrictions. As mentioned elsewhere, two of the lemmas demonstrated in [25] are key to accelerate the search of the optimal under constraints. It is a matter of future research to understand if our program can be used to accelerate the computation of the costs in the algorithm of [27].

2.3.1 Cost and variability among individual realizations of the strategy

The cost that the optimization seeks to minimize is an expected value. Any realization of the strategy, however, will result in a number of tests that will differ from the expected value. In order to study the variability among individual realizations of the nested strategy we performed stochastic numerical simulations as explained in the main

paper. Some of those results are in the paper. In this note we include two additional figures that serve to illustrate two aspects of the nested strategy. Fig. 3 shows that, in the optimal case, the probability that the samples of two infected individuals that are initially in the same pool remain in the same pool throughout the application of the strategy is not negligible.

We show in Fig. 3 histograms of the number of infected samples per pool for a stochastic simulation in which the strategy with $k = 3$, $m = (3^3, 3^2, 3)$, is applied to 1000 first-stage pools within which there are 544 infected samples (same simulation as in Fig. 3 (a) of the paper). We can observe that $\sim 6\%$ of the 1545 pools that are "tested" at the third-stage have two infected samples. The pools holding more than one infected sample at the k-th stage help are expected to reduce the average number of tests per individual needed to identify the infected ones. They therefore contribute to the inequality between the actual expected value of the number of tests per individual and the one where no more than one infected sample is present in each pool at all stages.

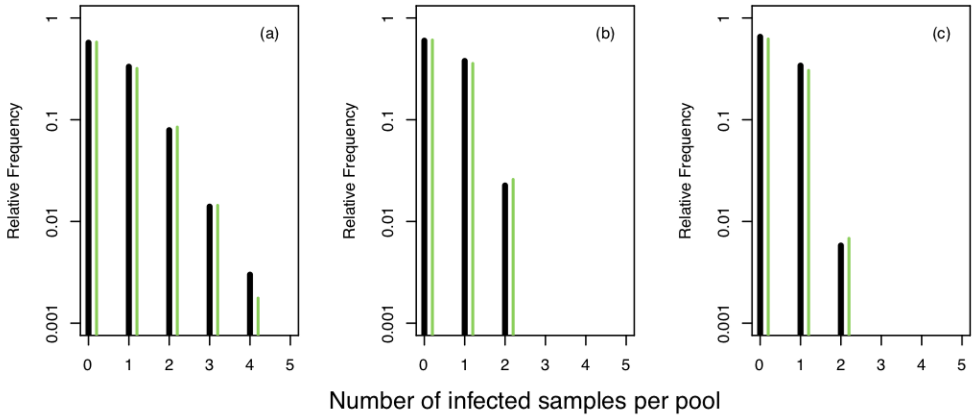

Fig 3. Histogram (as fraction of occurrences in log scale) of the number of infected samples per pool at the first (a), second (b) and third (c) stages for the same example as in Fig. 3(a) of the paper. Vertical axis in log-scale. Green bars correspond to the theoretical values computed as explained in Sec. 2.2.

We show in Fig. 4 (a) the results of stochastic numerical simulations in which we apply the strategy with $m = (3^k, \ldots, 3)$ and $k = 3$, which is optimal for $p \in\sim (0.01347, 0.03987)$, to a situation with infection prevalence, $\overline{p} = 0.00152$, (41 infected samples over 27000 analyzed). As before, the figure shows the histogram (in log scale and as fraction of occurrences) of the number of tests performed per initial pool obtained with 1000 numerical realizations of the strategy (*i.e.*, with 1000 initial pools of 27 samples each). We infer from the figure that, within these 1000 realizations, there are no instances in which the initial pool had more than one infected sample. The pools with exactly one infected sample are those requiring 10 tests to be resolved and the rest corresponds to pools with no infected samples and only one test. In this case the resulting average number of tests per individual, 0.0507, is equal to the one given by Eq. (22) with $\tilde{p} = \overline{p} = 0.00152$ and the resulting standard deviation is 0.066. We contrast this distribution with the one that is obtained with the strategy, $m = (3^k, \ldots, 3)$, that is optimal for $p \approx 0.0015$. We show in Fig. 4 (b) the histogram obtained with 1000 realizations of this strategy for which it is $k = 6$ (729,000 samples analyzed, 1133 infected). In this case, even if there are pools that need many more than 10 tests to be resolved, starting with a larger number of samples ($m_1 = 749$) results in a smaller average number of tests per individual, 0.0264, and standard deviation, 0.0212, than when using the suboptimal strategy with $k = 3$. If we restrict the number of realizations of the optimal strategy to 37, so that a similar number of individual

samples (27,713) is analyzed as in the 1000 realizations of the strategy with $k = 3$ (27,000), a smaller number of tests per individual (average number = 0.0256 with standard deviation= 0.0218) is obtained compared to that of the suboptimal case. However, in the analyzed example, the suboptimal case still produces a very noticeable reduction in the number of tests. Even when the resulting standard deviation, 0.066, is slightly larger than the expected value, 0.0507, it is clear that practically no pool will require more than 10 tests to be resolved (which is < 40% of the number of samples in the initial pool). In general, if we apply the $m = (\mu^k, \ldots, \mu)$ strategy to a situation with $p \ll p_{\mu,k+1}$, not only the expected number of tests per individual, $(1 + k\mu)/\mu^k$, will be given by Eq. (22), but more likely the number of tests per pool required will never exceed $(1 + k\mu)$, which is smaller than the number of individuals tested per initial pool, $m_1 = \mu^k$ for $k \geq 2$ and $\mu \geq 3$.

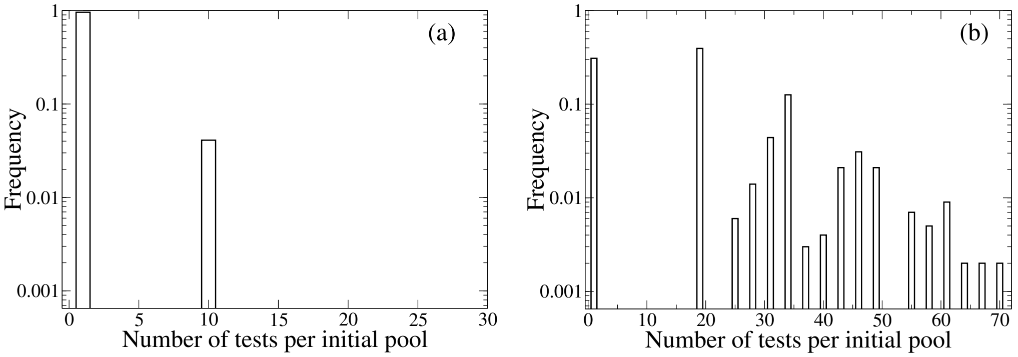

Fig 4. Histograms (as fraction of occurrences in log scale) of the number of tests performed per pool derived from 1000 numerical realizations in which strategies of the form $m = (3^k, \ldots, 3)$ are applied to a situation with infection prevalence, $\overline{p} = 0.00152$, using a sub-optimal, k in (a) ($k = 3$, 27,000 individual samples analyzed, 41 infected) and the optimal in (b) ($k = 6$, 729,000 samples analyzed, 1133 infected).

2.3.2 Algorithm to search for the optimal strategy when there are constraints

Although we have a prescription of the optimal nested strategy for every infection probability, $p > 2^{-51}$, sometimes there are constraints that prevent the optimal from being used. In such a case, an algorithmic approach can be used to search for the optimal strategy. The idea is to build a "tree" of sequences as described in what follows. To simplify the description we here characterize the (candidate) nested strategies by the sequence of numbers $m = (m_1, \ldots, m_k, m_{k+1})$ with $m_{k+1} = 1$, *i.e.*, with an additional 1 at the end that represents the stage of individual sample testing. The tree is then constructed as follows:

1. The root of the tree is the sequence with a single element, (m_1).
2. If the sequence $(m_1, \ldots, m_\ell)$ is a node of the tree and $m_\ell \neq 1$, there are as many branches emerging from this node as the number of proper submultiples of m_ℓ plus one. Here by proper we mean the natural numbers, $m_{\ell,1}, \ldots, m_{\ell,q}$, such that the ratios $m_\ell/m_{\ell,s}$, $1 \leq s \leq q$, are integers different from one. The nodes that branch out from $(m_1, \ldots, m_\ell)$ are: $(m_1, \ldots, m_\ell, m_{\ell,1}), \ldots (m_1, \ldots, m_\ell, m_{\ell,q})$, and $(m_1, \ldots, m_\ell, 1)$.
3. If the sequence $(m_1, \ldots, m_\ell)$ is a node of the tree and $m_\ell = 1$ there are no branches emerging from $(m_1, \ldots, m_\ell)$. This node then corresponds to a fesible nested strategy with $k = \ell - 1$.

As a consequence of this construction the terminal nodes of the tree are all the feasible sequences that start with the number, m_1, of the root. The cost, $D_k(m, p)$, of each of these sequences can be computed using Eq. (21). The sequence with the minimal value of $D_k(m, p)$ is the optimal for the given m_1 and p.

The number of nodes can be reduced by taking into consideration the results of two lemmas that were proved in [25]. Namely, that at an optimal solution with $m = (m_1, ..., m_k)$: *i)* the ratio m_j/m_{j+1} is not smaller than the ratio m_{j+1}/m_{j+2} for all $1 \leq j \leq k-1$; *ii)* m_j/m_{j+1} can only be equal to two for $j = k$. These two lemmas prune the tree and accelerate the search of the optimal strategy for a given m_1.

The algorithm based on this description is available at [26].

2.3.3 Practical implementations to reduce misclassification errors

The formulas derived so far, hold under the assumption that there are no testing errors. Facilities that identify virally infected individuals using RT-qPCR or related methods perform, simultaneously, several additional tests to reduce misclassifications. This is done even if samples are tested individually (*i.e.*, without pooling), for which the use of multi-well plates, as described elsewhere in the main paper and in this Supplementary file, is very convenient. We describe in what follows the errors that can be encountered, regardless of whether the samples are tested in pools or not, and the controls that are done to reduce these errors.

First of all, it is worth noticing that the probability of having false positives is very low. If they occur, on the other hand, it is very unlikely that they are not detected with the control tests. Namely, false positives may only occur if the sample is contaminated with nucleic acids of the virus or capable of giving a signal equal to that of the virus. If this contamination is introduced between the moment when the sample is extracted from the patient and the first opening of the tube in the lab (prior to the preparation of any pool), there is no way of knowing that the sample has been contaminated and will be identified as positive (unless, by chance, it is misclassified as negative). This, however, is not related to a solvable misclassification and would occur regardless of whether the test is run on pools or on individual samples. Laboratories take all the precautions not to have this contamination, so that these false positives are not observed in practice. A contaminant entering at any of the steps involved in the preparation and testing of the pools (or individual samples) would immediately be detected in today's automated systems in which the PCR test is run simultaneously on several tubes that have been prepared using the same master mix of reagents. To detect contaminations of this sort, it is mandatory that the testing facility, in all runs (regardless of whether the test is performed on pools or on individual samples) one or more negative controls be prepared and tested simultaneously with the tubes containing the patients' samples. The negative controls are tubes in which only a sample from uninfected people is added. Thus, if a negative control tube gives a positive result, the reagents are discarded and the entire run is processed again. It is highly unlikely that this type of contamination will occur at all in today's automated systems. The probability that it occurs, on the other hand, is independent of the infection probability and of whether the samples are tested in pools or individually. The number of tests that, on average, would have to be performed if some of them had to be repeated due to this contamination would be larger than the expected number by a similar factor if the pooling strategy were applied or if the samples were tested individually. Therefore, the optimal pooling strategy derived under the assumption that the tests carried no errors would give similar savings with respect to testing individual samples in the presence or not of this contamination.

The other aspect to be considered is the controls that are run to reduce the occurrence of false negatives (both for pool or individual testing). As described in what follows, there are many possible causes for the occurrence of false negatives. One of

them is the presence of an inhibitor of the PCR reaction in the testing tube. In order to detect the occurrence of this inhibition the internal control that is added consists in amplifying not only the RNA of the virus but also a human RNA that comes from the cells of the individual that are extracted to detect the infection. This is done simultaneously, in the same tube, using a master mix with the necessary components to identify both types of RNA. The result associated with the presence of the virus is considered negative only if it is seen that there is no signal for viral RNA but there is signal for human RNA in the same tube. As mentioned in the main body of the paper, when testing individual samples, if both signals are negative the result of the infection is not reported as negative or positive, as it may be due to the presence of inhibitors or because the sample was not well preserved or the RNA extraction failed. It is then recommended that a new sample be drawn from the patient. A similar approach should be followed if this problem is encountered when testing pools. If all the tests performed (including the positive control described in the following paragraph) happen to be negative for human RNA, it must be concluded that the reagents are deteriorated or the PCR machine is not working correctly.

Another possibility for a false negative is that the faulty component of the master mix is one of the virus-specific primers or the dye that is used to detect the products of the amplification that come from the viral RNA. In this case, the test would give a human RNA signal, but would fail to detect the viral RNA. In order to prevent this from happening, a "positive control" containing a specimen with viral and human RNA is tested simultaneously in the same run. A problem with the primers and/or the dye that gives the viral RNA signal will be noticed if this "positive control" tests negative for the viral RNA and positive for the human one.

The occurrence of false negatives due to the causes described so far can, in principle, be detected through the various controls that are mandatory to run in today's testing facilities. The probability of having faulty reagents or dyes is independent of the infection probability or of whether the tests are run on individual samples or on pools. The probability of having an inhibitor of the PCR in the testing tube, on the other hand, scales with the final volume that goes in the testing tube and is also independent of the number of samples or infection probability. Thus, the need of having to run some tests more than once due to the occurrence of these detectable errors would happen at the same rate for individual or pooled testing. Our optimal strategy, obtained under the assumption that there were no detection errors, would then give similar savings with respect to testing individual samples in this case as in the case with no detection errors. A similar situation holds for the problems that may arise during the RNA extraction procedure given that, for pool testing, this procedure is done on the pools.

In the case of pool testing there is an additional possibility for false negatives which we discuss in the manuscript: the internal (human RNA) control *per se* cannot detect the presence of a single or a few mishandled samples in a pool given that the human RNA coming from the "good" samples would give a detectable signal. The RNA quantification that ddPCR naturally provides at the end of the test can be used to solve this problem, as we describe in the main body of the paper. Thus, we have a detection method that would allow us to correct for this error.

Finally, another possible cause for false negatives is having samples with very low viral load. In that regard, performing tests on pools of samples exacerbates this problem, as the viral RNA content of each infected individual gets diluted when the sample is combined in a pool with the samples of others, particularly of people who are not infected. As shown in the main body of the paper, in 3-sample pools the presence of a single sample coming from an infected individual with the lowest viral load reported in [28] may be detected, according to our calculations (see Eq. (6) in the paper and Sec. 5 below), in more than 97% of the cases and, in more than 70%, if 9-sample pools

are used instead. The calculation shows that average viral loads can be detected even with 27-sample pools in over 99% of the cases. The studies of [29] show that the distribution of virions content among symptomatic or asymptomatic infected individuals is log-normal. Assuming that their estimate of virion concentration is equal to that of RNA copies and using the mean ($5.9\,10^6$virions/ml) and median ($2.5\,10^5$virions/ml) they obtained for one of their primers, we calculate that $\sim 0.5\%$ of the population has viral loads that, upon the $D = 8$ dilution of their experiments, would result in having, on average, one RNA copy or less in a $20\mu l$ testing volume ($\tilde{c}_{R;i}/D = 50/ml$). On the other hand, $\sim 93.5\%$ and $\sim 84\%$ of the infected population would have, respectively, $\tilde{c}_{R;i}/D > 700/ml$ and $\tilde{c}_{R;i}/D > 2500/ml$, allowing the detection of a single infected sample in pools of 3 or 9 samples with probability higher than 99%. This does not imply that only $\sim 92.6\%$ and $\sim 84\%$ of the infected population would be identified as infected when using 3 or 9-sample pools, respectively. Namely, when using the unrestricted optimal strategy for a given p, there is a non-negligible probability that two or more infected samples be contained in the same pool, especially at the first stage. This is illustrated by the stochastic simulations of Fig. 3. Having more than one sample corresponding to an infected individual in a pool would certainly facilitate the detection of the viral RNA in it. The numbers derived, on the other hand, depend on the assumption that the virion content estimated in [29] corresponds to RNA copies as measured by ddPCR. Comparing the calibration between Ct values of the RT-PCR test and virions/ml reported in [29] and the relationship between Ct values and RNA copies/test obtained with ddPCR in [28] we could estimate that the number of RNA copies/test of [28] is beween ~ 2.5 and 5 times that of virions derived in [29]. Using the 2.5 rescaling factor to go from virion [29] to RNA [28] content, we then obtain that 0.17% of the infected population described in [29] would have, on average, one RNA copy or less in a $20\mu l$ test and that 97%, 91.5% and 81.8% of the same population would have RNA number copies that would allow the detection of a single infected sample in pools of 3, 9 and 27 samples, respectively, with probability higher than 99%. These numbers, however, need a direct validation using ddPCR. It is worth noticing that the FDA in the US recommends that, after pooling, test performance should include $\geq 85\%$ positive agreement when compared with the same test performed on individual samples (`https://www.fda.gov/medical-devices/coronavirus-covid-19-and-medical-devices/pooled-sample-testing-and-screening-testing-covid-19#pooled`). Considering that the smallest viral load that is detected with ddPCR can be up to two orders of magnitude smaller than the lowest end detected with the current gold standard, RT-qPCR, it is likely that this 85% positive agreement between pool testing strategies using ddPCR and single sample testing will occur, especially if we restrict the maximum pool size to having ~ 27 samples. Analyzing in detail how all the aspects we have just described interact to determine the probability of detection in pools and in individual samples will be the subject of a future study.

2.3.4 Cost and test errors

Based on the previous descriptions, we analyze in this subsection the strategy under the assumption that there are no false positives, but that the presence of false negatives cannot be discarded.

Given $\ell \geq 1$, let us denote by $S_e(\ell)$ the probability a pool of ℓ samples is declared positive given that at least one sample in the pool is truly positive. In the absence of errors $S_e(\ell) = 1$ for all ℓ, and formula (3) computes the mean number of tests per individual associated to a nested strategy with $k + 1$ stages and sequence of pool sizes $m = (m_1, \ldots, m_k)$. When $S_e(\ell) < 1$ for some $\ell \geq 1$, the mean number of tests per

individual associated to the same strategy becomes

$$\frac{1}{m_1} + \sum_{j=2}^{k+1} \frac{\prod_{i=1}^{j-1} S_e(m_i)}{m_j} \big(1 - q^{m_{j-1}}\big) \tag{24}$$

as computed in [8], Section 6.3. Notice that the presence of these types of errors typically decreases the number of tests, this is due to the fact that a larger number of pools test negative, thereby stopping the search for infected samples in these pools. In terms of the mean number of tests per individual, the expression in (24) is less than or equal the associated expression in (3), for the same value of $q = 1 - p$, and the same strategy with parameters k, m.

The probability that an infected sample is identified as positive by the algorithm, on the other hand, can be computed as

$$S_e(k, m) = \prod_{j=1}^{k+1} S_e(m_j), \tag{25}$$

that is, the probability that at each stage j, with $1 \leq j \leq k+1$, the pool the sample belongs to is correctly identified as positive (see [8]).

Instead, Kim et al. [12] consider the mean number of false negative classifications per individual. For a nested strategy with parameters (m, k), this becomes

$$p\big(1 - S_e(k, m)\big) = p\Big(1 - \prod_{j+1}^{k+1} S_e(m_j)\Big). \tag{26}$$

Knowing the optimal strategy for a given p with and without constraints in the errorless case is also relevant when test errors are included. To illustrate this point, let us consider one of the examples discussed in [12]. In particular, in that paper the authors follow the recommendations of [30] which are based on results derived under the assumption that there is at most one infected sample per pool at any given stage, an assumption that we do not make. In particular, for the 3-step algorithm ($k = 2$ in our notation) without error that is considered in [12], this implies taking $m_2 = \sqrt{m_1}$. The subsequent analysis is then based on this choice. Let us then consider the case of identification of individuals with acute HIV infection discussed in Section 7 of [12]. In this case the authors consider the prevalence $p = 0.0002$, a 99% test specificity and a 90% test sensitivity and work under the assumption that the sensitivity and the specificity are independent of the total number of samples and infected samples in the pool. According to our calculations with no restrictions on the number of stages, the optimal for $p = 0.0002$ has 8 stages, the sequence of pool sizes is $m = (3^7, 3^6, ..., 3, 1)$, and the mean number of tests per sample is ≈ 0.0045, ignoring test errors. If, on the other hand, we limit the exploration to strategies having at most 3 stages, the optimal sequence of pool sizes given by our optimization program for this prevalence is $(307, 17, 1)$. In this case, if we also include the given specificity and sensitivity in the computation (see formula (3) in [12]), the mean number of tests per sample is approximately 0.01, which is better than 0.016, the mean number of tests per specimen associated to the strategy with pool sizes $(90, 10, 1)$ used in [12]. This implies that the number we can expect that would have to be performed using the strategy of [12] would be approximately $\sim 60\%$ larger than the number we can expect if our optimal strategy with constraints were used instead.

3 Estimating the infection probability, p.

The optimal strategy just described depends on the infection probability, p, a parameter that might be unknown *a priori*. As shown in the paper, applying a strategy that is optimal for a given p to a situation with smaller infection probability still produces a noticeable reduction in the number of tests per individual that have to be performed to identify the infected samples. The fact that PCR tests can be run in parallel on several (individual or pools of) samples at the same time using multi-well plates opens the possibility of applying a sub-optimal strategy and using the first round of tests to obtain an estimate of p. In this way, one option is to start with the strategy that is optimal for the largest p for which the pooling of samples is advisable ($p \sim 0.3$). The infected samples identified in this way could give an estimate of p with which to choose the strategy to be applied to the following batch of pools. Now, this largest p optimal strategy starts with pools of $m_1 = 3$ samples. Thus, if p is moderately small it is likely that, when running the strategy in parallel on a 96-well plate, no pool will test positive (~ 1.4 pools would test positive on average for $p = 0.005$). In such a case the first (parallel) run would not provide an estimate of p. Another possibility is to perform a first (parallel) round solely to estimate p. In either case, these runs would allow the identification of some of the non-infected samples (those that happen to be in pools with no infected samples). We describe in what follows how we can proceed if we choose one or the other option. In this work we focus on the use of the pool testing strategy to identify infected individuals. In the case of a pandemic, like the SARS-CoV-2 one, estimating the infection prevalence within the population of a certain region is also relevant to make decisions to prevent the spread of the epidemic. The procedures described in what follows can be used for this purpose as well. Since, for this application, it is a matter of estimating the probability, and not of producing correct identifications for all individuals, the possibility of false negatives or positives has no significant effect. The probability estimate, p, would be obtained at a much cheaper cost with essentially the same effectiveness as if samples were tested individually.

3.1 Running a set of parallel tests to infer p.

Let us assume that we are working with a 96-well plate. Situations with plates of other sizes can be handled analogously.

1. We fill 32 pools with $m_1 = 3$ samples, another 32 pools with $m_1 = 9$ and the last 32 with $m_1 = 27$ samples.

2. We run the test on the 96 pools simultaneously and determine the number, W_h, of pools with $m_1 = h$ ($h = 3, 9, 27$) that test positive in this first run.

3. We compute the estimator of the probability, $\hat{P}_h$, that a pool with $m_1 = h$ samples within a set of $N_w = 32$ pools turns out to be positive as:

$$\hat{P}_h = \frac{W_h}{N_w + \frac{h-1}{2h}}. \tag{27}$$

4. We derive an estimator, $\hat{p}$, of the (individual) sample infection probability, p, as:

$$\hat{p} = \frac{1}{3}\left(\hat{p}_3 + \hat{p}_9 + \hat{p}_{27}\right), \tag{28}$$

where

$$\hat{p}_h = 1 - (1 - \hat{P}_h)^{1/h}; \quad h = 3, 9, 27. \tag{29}$$

The term $\frac{h-1}{2h}$ is added in order to reduce the bias [31]. If any of the values, $\hat{p}_h$, is too small, it is best to follow the procedure described in the next section.

3.2 Dynamic estimation of p as the strategy is applied

In this case we apply the strategy as stated in the manuscript, with some modifications depending on the outcome of the first set of parallel tests. If p is completely unknown but one wants to apply the strategy anyway, it is best to start with $m_1 = 3$. The test is then run simultaneously on 96 pools with 3 samples each. There are four possible types of outcomes and, thus, of subsequent steps, as described in what follows.

I) *If the number of 3-sample pools that test positive in the first run, N_p^+, is such that $0 < 3N_p^+ < 96$:*

1. We obtain an estimate, $\hat{p}$, of the individual sample infection probability as $\hat{p} = \hat{p}_h$ using Eqs. (27) and (29) with $N_w = 96$, $h = 3$ and $W_h = N_p^+$. We derive the optimal pool size for the estimated probability, $m_1(\hat{p})$, as:

$$m_1(\hat{p}) = 3^{\hat{k}_3}, \text{with} \quad \hat{k}_3 = \Big\lfloor \log_3\Big(\frac{1}{\log_3(1/(1-\hat{p}))}\Big)\Big\rfloor. \tag{30}$$

2. We accommodate, in the 96-well plate, the $3N_p^+$ samples of the N_p^+ pools that tested positive in the first stage and $96 - 3N_p^+$ pools with $m_1 \leq m_1(\hat{p})$ samples each. If $m_1(\hat{p})$ is smaller than the maximum number allowed for a reliable detection, then we set $m_1 = m_1(\hat{p})$, if not we choose the largest number of the form $m_1 = 3^k$ that allows the reliable detection of one infected sample in an m_1-pool.

3. After the second run of 96 parallel tests is done we know the number, N_i, of infected samples that were contained in the first set of 96 3-sample pools. We can then obtain a better estimate of the infection probability as:

$$\hat{p} = \frac{N_i}{96h}, \tag{31}$$

   with $h = 3$. Using this estimate we recompute the best pool size, m_1, using Eq. (30).

4. For the third run of tests, we use as many wells as necessary to accommodate the $(m_1/3)$-sample pools that come from the m_1-sample pools that tested positive in the second run and, if there is room, pools with the newly determined value of m_1. This procedure is then continued as long as there are samples.

II) *If the number of 3-sample pools that test positive in the first run, N_p^+, is such that $96 \leq 3N_p^+ < 3 \cdot 63 = 189$:*

1. We obtain an estimate, $\hat{p}$, of the individual sample infection probability as in the previous case, namely using Eqs. (27) and (29) to compute $\hat{p}_h$ with $h = 3$ and then set $\hat{p} = \hat{p}_h$. We then use Eq. (30) to estimate the best pool size, $m_1(\hat{p})$.

2. We test individually 96 of the $3N_p^+$ samples contained in the N_p^+ pools that tested positive in the first run. We keep on doing this with the other individual samples of the initial N_p^+ pools as long as there are no unused wells in the plate. Once there is room, we pool the new samples in groups of m_1 samples each with m_1 given by the estimate, $m_1(\hat{p})$, derived in the previous step if this value does not exceed the maximum allowed for a reliable detection. Otherwise, we choose, as before, the largest $m_1 = 3^k$ that satisfies the restriction for a reliable detection.

3. Once all the samples contained in the initial 3-sample pools are identified as infected or not, we know the number, N_i, of infected samples in the initial batch with which we can obtain a better estimate of the infection probability using Eq. (31) with $h = 3$ and then compute the best pool size, m_1, with Eq. (30).

III) *If the number of 3-sample pools that test positive in the first run, N_p^+, is such that $3N_p^+ \geq 3 \cdot 63 = 189$:*

1. We continue working with individual samples (no pooling) both with those contained in the N_p^+ pools that tested positive in the first run and with the untested samples so far. For the latter, each run will give the number of infected individuals, N_i, from which the infection probability, $\hat{p}$, can be estimated using Eq. (31) with $h = 1$. As long as the obtained value satisfies $\hat{p} \geq 1 - 1/3^{1/3} \approx 0.307$ we continue without pooling. Otherwise, the optimal m_1 can be determined with Eq. (30) and a strategy that starts with this value of m_1 (or smaller as discussed before) is applied.

IV) *If none of the 3-sample pools tests positive:*

1. We choose m_1 as the the maximum number of the form $m_1 = 3^k$ that allows the reliable detection of one infected sample within an m_1 pool.

If an estimate, $\hat{p}$, of the infection probability is available *a priori*, we then start with pools of size m_1 as defined in Eq. (30). If only the order of magnitude of $\hat{p}$ is known, we then start with $m_1 = 3^{\hat{k}_3 - 1}$ with $\hat{k}_3$ as in Eq. (30). After the first run of tests is performed on the 96 pools containing m_1 samples each, we compute the estimator, $\hat{P}_h$, of the probability that a pool tests positive using Eq. (27) with $N_w = 96$ and $h = m_1$. The individual sample probability is then estimated using Eq. (29) with $h = m_1$. This newly determined estimate, $\hat{p}$, is then used to decide the size of the new pools if there is room to place them in the plate together with those that come from the 96 pools tested in the first run, as explained in II) before. Once the number, N_i, of infected samples among the $96m_1$ tested in the first run is known, a new estimate of the infection probability can be obtained using Eq. (31) with $h = m_1$. The infection probability can be re-estimated as the strategy is applied either using Eqs. (27) and (29) or Eq. (31), depending on whether we use the information drawn, respectively, from the first or the last stage of the strategy. Averaging the estimates obtained in various ways could also be a good option.

4 Estimating nucleic acid content using ddPCR

4.1 The general case

In ddPCR the volume of each test, *i.e.*, the testing volume, V_t, that goes into one well is divided into M sub-volumes, V_{gt}, of $\sim 1nl$ each:

$$V_t = MV_{gt}. \tag{32}$$

M varies depending on the equipment. We will use $M = 20,000$, $V_{gt} = 1nl$ and $V_t = 20\mu l$. The testing volume, V_t, in turn, comes from a dilution of a volume, V_s, that is taken from the sample. Namely, V_t contains the nucleic acid molecules contained in a volume, V_s, taken from the original sample and other solutions with the material that is needed for the test to proceed. Given that this procedure does not add any new molecules of the nucleic acids that the test identifies, for the purpose of their quantification we merely think of it as introducing a dilution of factor, D, so that:

$$V_t = DV_s. \tag{33}$$

V_s, on the other hand, is part of the volume of biological material that is taken from the individual to detect the presence of the nucleic acids of interest. We call, V_o, the volume

of this biological material of which V_s is a sub-volume with no dilutions involved. We clarify what we mean by this in what follows. The aim of the quantification is to determine the concentration, c_R, of the nucleic acids in V_o. To this end, we think of V_s as the sum of $M = 20,000$ sub-volumes, V_{gs}, each of which, when diluted by the factor D, corresponds to one of the sub-volumes of the ddPCR test:

$$V_s = MV_{gs}, \tag{34}$$
$$V_{gt} = DV_{gs}. \tag{35}$$

We assume that each of the sub-volumes, V_{gs}, is a sufficiently small fraction of V_o so that the numbers of nucleic acid molecules contained in each of them, N_{Rg}, are independent identically distributed (i.i.d.) random variables. The mean of these variables is such that

$$\langle N_{Rg} \rangle = c_R V_{gs} = \frac{c_R}{D} V_{gt}. \tag{36}$$

The first of the two equalities in Eq. (36) reflects what we meant before when we said that there was no dilution involved in the extraction of V_s from V_o. Given that the dilution that transforms V_s into V_t does not change the nucleic acid content, these i.i.d. random variables also correspond to the numbers of nucleic acid molecules in each of the tested sub-volumes, V_{gt}. The variables, N_{Rg}, are described by a Binomial distribution:

$$N_{Rg} \sim B(N_{Ro}, p_{gs}), \tag{37}$$

of parameters

$$N_{Ro} = c_R V_o, \quad p_{gs} = \frac{V_{gs}}{V_o} = \frac{V_{gt}}{DV_o}. \tag{38}$$

As we show later, the Poisson approximation with mean given by Eq. (36) serves very well for our purpose too. ddPCR is an end-point method which determines (ideally) how many of the M $V_{gt} \sim 1nl$ sub-volumes contained at least one nucleic acid molecule. Under the assumption that the M numbers, N_{Rg}, are i.i.d. according to Eqs. (37)-(38), the fraction of sub-volumes with at least one nucleic acid molecule is an estimator of the probability, P_{+g}, that one such volume contains at least one of these molecules:

$$P_{+g} = 1 - P(N_{Rg} = 0) = 1 - (1 - p_{gs})^{N_{Ro}}. \tag{39}$$

Using the second equation in (38) we can rewrite Eq. (39) as:

$$P_{+g} = 1 - \left(1 - \frac{V_{gs}}{V_o}\right)^{N_{Ro}}, \tag{40}$$

or, equivalently, as:

$$\log\left(1 - \frac{V_{gs}}{V_o}\right) = \frac{1}{N_{Ro}} \log\left(1 - P_{+g}\right). \tag{41}$$

Given Eq. (34) and the fact that $V_s < V_o$ it is $V_{gs}/V_o < 1/M = 5\,10^{-5}$, the l.h.s. of Eq. (41) can be approximated by $-V_{gs}/V_o$. With this approximation and using the two equations in (38), Eq. (41) can be rewritten as:

$$c_R \frac{V_{gt}}{D} = -\log\left(1 - P_{+g}\right). \tag{42}$$

This equation is also obtained if the variables, N_{Rg}, are assumed to be Poisson distributed with mean given by Eq. (36) in which case $1 - P_{+g} = \exp(-c_R V_{gt}/D)$.

Let us assume that the test determined that M_+ of the M sub-volumes contained molecules of the nucleic acid of interest. As explained in Sec. 4.3, if $MP_{+g} > 5$ and $M(1 - P_{+g}) > 5$, the fraction:

$$\hat{P}_{+g} = \frac{M_+}{M}, \tag{43}$$

is an estimator of P_{+g} such that:

$$P_{+g} \in \left[\hat{P}_{+g} - 1.96\sqrt{\frac{\hat{P}_{+g}\left(1 - \hat{P}_{+g}\right)}{M}}, \hat{P}_{+g} + 1.96\sqrt{\frac{\hat{P}_{+g}\left(1 - \hat{P}_{+g}\right)}{M}} \right], \tag{44}$$

with 95% confidence. If not, other intervals for P_{+g} can be estimated as explained in Sec. 4.3. The "large sample" limit ($MP_{+g} > 5$ and $M(1 - P_{+g}) > 5$) guarantees that the borders of the interval are bounded away from 0 and 1. Another important property of this limit is that $\hat{P}_{+g}$ is normally distributed around P_{+g}. We can then replace P_{+g} in Eq. (42) by its estimator, $\hat{P}_{+g}$, [32] and obtain an estimate, $\hat{c}_R$, of the nucleic acid concentration in the original sample, c_R, as:

$$\hat{c}_R \frac{V_{gt}}{D} = -\log\left(1 - \hat{P}_{+g}\right). \tag{45}$$

This is the usual formula with which the content of nucleic acids is quantified in ddPCR experiments (see *e.g.*, [32]). In view of Eq. (44), the range of possible values, c_R, for each value of $\hat{P}_{+g}$ is then given by:

$$-\log\left(1 - \hat{P}_{+g} + 1.96\sqrt{\frac{\hat{P}_{+g}\left(1 - \hat{P}_{+g}\right)}{M}}\right) \leq c_R \frac{V_{gt}}{D}$$
$$\leq -\log\left(1 - \hat{P}_{+g} - 1.96\sqrt{\frac{\hat{P}_{+g}\left(1 - \hat{P}_{+g}\right)}{M}}\right). \tag{46}$$

Fig. 5 illustrates how the range of c_R values given by Eq. (46) and the correspondig relative uncertainty behave as functions of $\hat{P}_{+g} = M_+/M$ for values of $\hat{P}_{+g}$ for which the large sample limit holds as described in what follows.

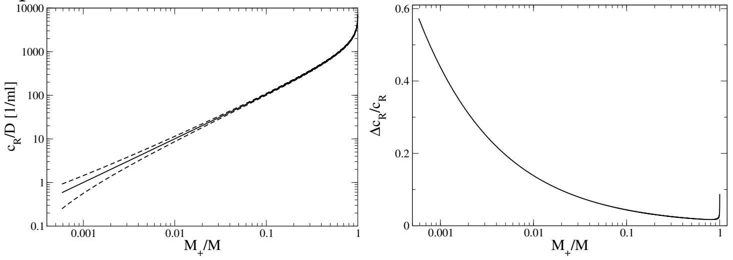

Fig 5. (a) Predictor, $\hat{c}_R/D$, of the nucleic acid concentration in the testing well derived from Eq. (45) (solid line) and upper and lower limits of the values that the same concentration, c_R/D, can on with a 95% confidence level derived from Eq. (46) (dashed lines) as functions of the experimentally determined, $\hat{P}_{+g} = M_+/M$, for $\hat{P}_{+g}$ within the range defined in Eq. (47). For all computations we used $M = 20,000$ and $V_{gt} = 10^{-3}ml$. (b) Relative uncertainty of the predicted nucleic acid concentration, c_R/D, (*i.e.* one half of the difference between the upper and lower limits plotted with dashed lines in (a) divided by the predicted value, $\hat{c}_R/D$, of Eq. (45))as a function of $\hat{P}_{+g} = M_+/M$.

Given Eq. (44), the conditions for the large sample limit to hold, $MP_{+g} > 5$ and $M(1-P_{+g}) > 5$, are satisfied if $\left(\hat{P}_{+g} - 1.96\sqrt{\hat{P}_{+g}(1-\hat{P}_{+g})/M}\right) > 5/M$ and $\left(1 - \hat{P}_{+g} - 1.96\sqrt{\hat{P}_{+g}(1-\hat{P}_{+g})/M}\right) > 5/M$ from which we obtain:

$$5.85\,10^{-4} < \hat{P}_{+g} < 0.999415. \tag{47}$$

This implies that the large sample limit can be used if the number of sub-volumes that test positive satisfy $11 < M_+ < 19989$. In terms of the estimated concentration, Eq. (47) can be rewritten as:

$$\frac{5.85\,10^{-4}}{V_{gt}} < \frac{\hat{c}_R}{D} < \frac{7.44}{V_{gt}}, \tag{48}$$

or, equivalently, that

$$\frac{11.7}{V_t} < \frac{\hat{c}_R}{D} < \frac{1.49\,10^5}{V_t}, \tag{49}$$

$$\frac{585}{ml} < \frac{\hat{c}_R}{D} < \frac{7.4\,10^6}{ml}, \tag{50}$$

where we have used that the testing sample, $V_t = 20\mu l$, is such that $V_t = 20,000V_{gt}$. As discussed in the paper, these ranges allow the quantification of viral RNA in most of the samples taken from people infected with SARS-CoV-2, with the exception of those with the very highest viral loads [23, 33–35]. As illustrated in Fig. 5, the uncertainty in the determination of the concentration, on the other hand, remains at manageable levels throughout the range for which the large sample limit holds. Even though at very low values the uncertainty is $\sim 60\%$, which implies that only its order of magnitude can be determined when $c_R/D \sim \mathcal{O}(10^{-1}/ml)$, at $M_+/M \sim 0.005$ the uncertainty is already of the order of 20%. For larger, M_+/M, the uncertainty goes down and never exceeds 10%.

4.2 The case of pooled samples.

Let us apply now the analysis just described to the case of pools of samples. To this end, let us denote by *original sample* the sample that is taken from an individual before it is diluted and/or mixed with any other sample to proceed with the test. Let us identify each of the original samples that go in the pool of interest with the subscript, i, $1 \le i \le m_j$. Each of these original samples has a volume, $V_{o;i}$ and a certain concentration, $\tilde{c}_{R;i}$, of the RNA that is to be detected by the test ($\tilde{c}_{R;i} = 0$ is an option). Let us assume that, for the test, a volume, $V_{s;i}^{(j)}$, is drawn from $V_{o;i}$ for each $1 \le i \le m_j$. We assume that the volume extraction is done in such a way that guarantees a small variability of $V_{s;i}^{(j)}$ among samples so that $V_{s;i}^{(j)}$ has a fixed value, $V_s^{(j)} = V_s/m_j$, for all values of i. The volumes, $V_{s;i}^{(j)}$, $1 \le i \le m_j$, are at some point combined into one volume, V_s, and diluted with other volumes that contain the materials for the test to proceed. We call this final volume the testing volume, $V_t \sim 20\mu l$. These various volumes are then related by:

$$V_t = DV_s = D\sum_{i=1}^{m_j} V_{s;i}^{(j)} = m_j D V_s^{(j)}, \tag{51}$$

where D is the dilution factor. Given that $V_{s;i}^{(j)} \ll V_{o;i}$, the number of RNA molecules in $V_{s;i}^{(j)}$ is a Poisson-distributed random variable, $\mathcal{N}_{R;i}^{(j)}$ of mean:

$$E\mathcal{N}_{R;i}^{(j)} = \tilde{c}_{R;i} V_{s;i}^{(j)} = \tilde{c}_{R;i} V_s^{(j)}. \tag{52}$$

The total number of RNA molecules in the m_j-sample pool that is tested is then:

$$N_R^{(j)} = \sum_{i=1}^{m_j} \mathcal{N}_{R;i}^{(j)}. \tag{53}$$

$N_R^{(j)}$ is Poisson distributed with mean:

$$EN_R^{(j)} = V_s^{(j)} \sum_{i=1}^{m_j} \tilde{c}_{R;i} = V_s \frac{1}{m_j} \sum_{i=1}^{m_j} \tilde{c}_{R;i}. \tag{54}$$

Thus, the concentration that can be estimated in the case of an m_j-sample pool is:

$$c_R^{(j)} \equiv \frac{1}{m_j} \sum_{i=1}^{m_j} \tilde{c}_{R;i}. \tag{55}$$

Let us now consider an m_j-sample pool with $m_j > 1$ that tests positive at the j-th stage so that, at stage $j+1$, it is sub-divided into m_j/m_{j+1} sub-pools with m_{j+1} samples each. Given Eq. (51), the size of the volumes that are extracted from the original samples at this stage, $V_s^{(j+1)}$, are related to the previously defined volumes by:

$$V_t = DV_s = m_j DV_s^{(j)} = m_{j+1} DV_s^{(j+1)}. \tag{56}$$

The number of RNA molecules contained in the volume, $V_{s;i}^{(j+1)}$, extracted from the i-th original sample is again a Poisson-distributed random variable, $\mathcal{N}_{R;i}^{(j+1)}$, with mean:

$$E\mathcal{N}_{R;i}^{(j+1)} = \tilde{c}_{R;i} V_s^{(j+1)}. \tag{57}$$

The number of RNA molecules in each of the m_{j+1}-sample sub-pools is then:

$$N_{R;\ell}^{(j+1)} = \sum_{i=1+(\ell-1)m_{j+1}}^{\ell m_{j+1}} \mathcal{N}_{R;i}^{(j+1)}, \qquad 1 \leq \ell \leq \frac{m_j}{m_{j+1}}, \tag{58}$$

and, as in Eqs.(54)–(55), their means satisfy:

$$EN_{R;\ell}^{(j+1)} = V_s c_{R;\ell}^{(j+1)} \equiv V_s \frac{1}{m_{j+1}} \sum_{i=1+(\ell-1)m_{j+1}}^{\ell m_{j+1}} \tilde{c}_{R;i}, \tag{59}$$

where $c_{R;\ell}^{(j+1)}$ is the RNA concentration that is estimated for the ℓ-th sub-pool at the end of the ($j+1$-stage) ddPCR test. The sum of the RNA molecules over all the sub-pools that come from the same m_j-sample pool is:

$$N_R^{(j+1)} = \sum_{\ell=1}^{m_j/m_{j+1}} N_{R;\ell}^{(j+1)} = \sum_{i=1}^{m_j} \mathcal{N}_{R;i}^{(j+1)}, \tag{60}$$

and, given Eqs. (56), (57), (59) and (60), its mean satisfies:

$$EN_R^{(j+1)} = V_s \sum_{\ell=1}^{m_j/m_{j+1}} c_{R;\ell}^{(j+1)} = V_s^{(j+1)} \sum_{i=1}^{m_j} \tilde{c}_{R;i} = V_s \frac{1}{m_{j+1}} \sum_{i=1}^{m_j} \tilde{c}_{R;i}. \tag{61}$$

Eqs. (55) and (61) imply that:

$$\sum_{\ell=1}^{m_j/m_{j+1}} c_{R;\ell}^{(j+1)} = \frac{m_j}{m_{j+1}} c_R^{(j)}, \tag{62}$$

which, as explained in the paper, can be used to check the self-consistency of the results.

4.3 Formulas to estimate probability from fraction of occurrences.

Here we compile some well established results on the estimation of probabilities from fraction of occurrences, including a discussion on the conditions that guarantee the validity of Eq. (44). Let us assume that an observation can fall into one of two categories, + and −, with probabilities, P and $Q = 1 - P$, respectively. Let us assume that, given M independent observations, a fraction $\hat{P}$, of them falls in the + category. Then, if M is large enough, $\hat{P}$ is a point estimator of P such that:

$$P \in \left[\hat{P} - \zeta_{\alpha/2}\sqrt{\frac{\hat{P}\left(1-\hat{P}\right)}{M}}, \hat{P} + \zeta_{\alpha/2}\sqrt{\frac{\hat{P}\left(1-\hat{P}\right)}{M}}\right], \tag{63}$$

with a $100(1-\alpha)$ confidence level (*i.e.*, there is at least a $100(1-\alpha)\%$ chance that P is contained in the interval given by Eq. (63)) where $\zeta_{\alpha/2}$ is the critical value of the normal distribution for the confidence level [36, 37]. In particular, for $\alpha = 0.05$ (95% confidence) it is $\zeta_{\alpha/2} = 1.96$ and for $\alpha = 0.01$ (99% confidence) it is $\zeta_{\alpha/2} = 2.58$. The large sample approximation holds if $MP \geq 5$ and $MQ \geq 5$ [37] and is most accurate if $0.3 \leq P \leq 0.7$ [36]. Furthermore, under this approximation, the fraction $\hat{P}$ is normally distributed with mean, P, and standard deviation, $\sigma_P = \sqrt{PQ/M}$. The confidence interval is sometimes enlarged to include a correction for continuity by adding (subtracting) $1/(2M)$ to the upper (lower) limit of the interval in Eq. (63) [37]. Outside this limit, more complicated expressions can be used for the lower, P_L, and upper, P_U, limits of the confidence interval that are always valid for a binomially distributed variable:

$$P_L = \frac{x}{x + (M-x+1)F_{2(M-x+1),2x;\alpha}}, \tag{64}$$

$$P_U = \frac{(x+1)F_{2(x+1),2(M-x);\alpha}}{M - x + (x+1)F_{2(x+1),2(M-x);\alpha}}, \tag{65}$$

with $x = \hat{P}M$ and where the $F_{a,b;\alpha}$ is the $100(1-\alpha)$th percentile of the F distribution with two degrees of freedom [37]. If $MP \geq 5$ and $MQ \geq 5$ but $P < 0.3$ or $P > 0.7$, the lower and upper limits can be computed as:

$$P_L = \frac{2M\hat{P} + \zeta_{\alpha/2}^2 - 1 - \zeta_{\alpha/2}\left(\zeta_{\alpha/2}^2 - \left(2 + \frac{1}{M}\right) + 4\hat{P}\left(M\hat{Q} + 1\right)\right)^{1/2}}{2\left(M + \zeta_{\alpha/2}^2\right)}, \tag{66}$$

$$P_U = \frac{2M\hat{P} + \zeta_{\alpha/2}^2 + 1 + \zeta_{\alpha/2}\left(\zeta_{\alpha/2}^2 + \left(2 - \frac{1}{M}\right) + 4\hat{P}\left(M\hat{Q} + 1\right)\right)^{1/2}}{2\left(M + \zeta_{\alpha/2}^2\right)}, \tag{67}$$

with $\zeta_{\alpha/2}$ as defined before and $\hat{Q} = 1 - \hat{P}$.

5 Minimum viral load detectability and maximum pool size using ddPCR

The description introduced in the previous subsection allows us to determine the maximum pool size for which the presence of a single infected sample can be detected with a certain probability. Assuming that ddPCR is able to detect even a single RNA

particle, then the presence of only one infected sample in an m_j-sample pool will be detected if there is at least one viral RNA molecule in the volume of size, V_s/m_j, with which the sample of the infected individual contributes to the total sample volume, V_s. Let us assume that the only infected sample of the m_j-pool is the i-th one. Using the notation and assumptions of the previous subsection, the probability that there is at least one viral RNA molecule in the testing volume is:

$$P(N_R^{(j)} \geq 1) = P(\mathcal{N}_{R;i}^{(j)} \geq 1) = 1 - \exp\left(-\tilde{c}_{R;i}\frac{V_s}{m_j}\right). \tag{68}$$

Setting a lower bound to this probability we can determine the minimum detectable viral concentration for a given m_j or, conversely, the maximum pool size that allows the detection of at least a certain viral RNA concentration. Inserting Eq. (33) in Eq. (68) we obtain the expression that we use in the paper.

6 Computation of likelihoods for the detection of flawed samples in pools.

The viability of the tested sample and the quality of the amplification procedure are checked by amplifying the genetic material associated to a piece of human RNA that should be present in any good sample. As described in the paper, we are interested in the possibility of identifying the presence of a single flawed sample in an m-sample pool on which a ddPCR test is run. The underlying assumption is that the conditions that guarantee that this distinction is possible would also allow the identification of cases with more than one flawed sample. To this end, we assume that the number of RNA molecules of the i-th individual in a volume of size, V_s, drawn (with no dilution) from the original sample of the same individual is described by a Poisson distribution with mean λ_i. The distribution is Poisson as well for any sub-volume of V_s but with mean rescaled by the ratio of volume sizes. Thus, when pooling together m samples whose mean numbers of human RNA molecules in V_s are, respectively, $\lambda_1, \lambda_2, \ldots, \lambda_m$, the number of RNA molecules in V_s of the pool is a Poisson distributed random variable of mean, $\sum_i \lambda_i/m$. To proceed with the calculation we need to consider the distribution of the human RNA content within the human population. Now, the human RNA content (as well as the viral one) contained in the samples that arrive at the testing facility depends on multiple factors including the type of specimen (swabs, saliva, others), the sample handling procedures, the RNA extraction methods, reagents supplier and PCR calibration, besides the intrinsic variability among individuals. There is no empirical data that can support a particular choice for the distribution of human RNA in the samples that are tested. We then decided to work under the most parsimonious hypothesis which is to assume that this content follows a Normal distribution, *i.e.*, that the individual means, λ_i, of the good samples are actually instances of a Normal distributed random variable of mean λ and standard deviation, σ. The results, however, are not very sensitive to this hypothesis. In fact, if instead we consider an exponential distribution with the same mean, since the important variable is the sum of the $\lambda_i s$, the central limit theorem (at least for $m \gtrsim 20$) ensures that the approximation is adequate. We also assume that flawed samples have $\lambda_i = 0$. Under these assumptions, an m-sample pool is characterized by a sequence, $\lambda_1, \lambda_2, \ldots, \lambda_m$ of i.i. (Normal) distributed random variables, if all the samples are good. If there is one flawed sample in the pool, then, the corresponding λ_i will be zero and the other $m - 1$ will be i.i.d. random variables. We notice that, in both cases, the number of RNA molecules is the same in V_s and in the test volume, $V_t = DV_s$, because the dilution does not change the relevant RNA content.

We now consider that we run the ddPCR test on the pool (subdividing the test volume, V_t, into M sub-volumes) and that the number of sub-volumes (nano-droplets) containing no human RNA, X, is equal to x_{obs}. The two situations that we want to distinguish are: (i) all the individual samples were "good", *i.e.* had $\lambda_i \neq 0$; (ii) one of the samples was "flawed", *i.e.* had $\lambda_i = 0$. We work under the assumption that, if a test satisfies the constraints which guarantee that these two situations are distinguishable, then tests with more than one flawed sample will also be distinguishable from "good" tests. For the comparison we first compute the likelihood of observing $X = x_{obs}$ under both hypotheses. We then compare the likelihoods and compute the critical value, x_c, that establishes the border between having one or the other hypothesis as the more probable one given the observations. Finally we compute the accuracy of the classification, *i.e.*, the fraction of instances for which the classification is correct. Given the values, $\lambda_1, \lambda_2, \ldots, \lambda_m$, the probability that there is no RNA in a nano-droplet is $p_{n-f} = \exp(-\sum_i \lambda_i/(Mm))$. Under the assumption that $\lambda_1, \lambda_2, \ldots, \lambda_m$ is a sequence of iid random variables with distribution, $Normal(\lambda, \sigma)$, then $p_{n-f} = e^{-\frac{1}{Mm}Z}$ with $Z \sim Normal(m\lambda, \sqrt{m}\sigma)$. If $m-1$ of the λ_is are Normal and one of them is zero, then $p_{n-f} = e^{-\frac{1}{Mm}Z}$ with $Z \sim Normal((m-1)\lambda, \sqrt{m-1}\sigma)$. Therefore, the likelihoods of obtaining $X = x_{out}$ in each of these situations are:

$$\begin{aligned} P(X = x_{obs}|\text{all good}) &= \int_{-\infty}^{\infty} \binom{M}{x_{obs}} e^{-\frac{z}{Mm}x_{obs}} (1 - e^{-\frac{z}{Mm}})^{M-x_{obs}} \phi_{m\lambda,\sqrt{m}\sigma}(z)dz \\ &\approx \binom{M}{x_{obs}} e^{-\lambda x_{obs}/M} (1 - e^{-\lambda/M})^{M-x_{obs}}, \qquad (69) \\ P(X = x_{obs}|\text{one flawed}) &= \int_{-\infty}^{\infty} \binom{M}{x_{obs}} e^{-\frac{z}{Mm}x_{obs}} (1 - e^{-\frac{z}{Mm}})^{M-x_{obs}} \phi_{(m-1)\lambda,\sqrt{m-1}\sigma}(z)dz \\ &\approx \binom{M}{x_{obs}} e^{-\lambda(1-1/m)x_{obs}/M} (1 - e^{-\lambda(1-1/m)/M})^{M-x_{obs}}, \qquad (70) \end{aligned}$$

where $\phi_{\mu,\sigma}(z) = \frac{1}{\sqrt{2\pi}\sigma} e^{\frac{(x-\mu)^2}{2\sigma^2}}$ and $\binom{M}{x_{obs}}$ is the corresponding binomial coefficient. Given that the two likelihoods are approximately equal to Binomial distributions centered at different values ($Me^{-\lambda/M}$ and $Me^{-\lambda(1-1/m)/M}$), the threshold, x_c, can be derived by equating the r.h.s. of Eqs. 69 and 70. We obtain:

$$x_c = M\left(1 - \frac{1}{1 - \frac{mM}{\lambda}\left(\log\left(1 - e^{-\lambda(1-1/m)/M}\right) - \log\left(1 - e^{-\lambda/M}\right)\right)}\right). \qquad (71)$$

If the Binomial distributions can be approximated by Normal ones [31], then a high accuracy is guaranteed if $P(X \leq x_c|\text{one flawed}) \approx P(X \geq x_c|\text{all good})$. Using the normal approximation, the accuracy can then be written as:

$$\text{Accuracy} = \Phi\left(\frac{x_c - Me^{-\lambda/M}}{\sqrt{M(1 - e^{-\lambda/M})e^{-\lambda/M}}}\right), \qquad (72)$$

where $\Phi(z) = \int_{\infty}^{z} \phi_{0,1}(x)dx$ with $\phi_{\mu,\sigma}(z)$ as defined before. In the paper we show plots of the accuracy for different pool sizes. It is important to notice that the Normal approximation of the Binomial distribution that led to Eq. (72) does not have a noticeable impact on the "relevant part" of the accuracy. Namely, this approximation is not accurate only when $\exp(-\lambda/M)$ is near one or zero, or, equivalently, when the mean human RNA, λ, is almost zero or huge. The human RNA that is used as an internal control, however, is chosen so that it can be detectable (*i.e.*, with content that is bounded away from zero) and within the limits of the PCR dynamic range (*i.e.*, not

huge). Nevertheless, in these two particular limits, the accuracy can be calculated exactly using the Binomial distributions and it is equal to 1/2 as shown in Fig. 6(b) of the main body of the paper.

7 Running the strategy in parallel. Numerical examples.

We show in Tables 1 and 2 the results of stochastic numerical simulations performed as before but this time assuming that at most 96 tests can be run simultaneously and that at most 32 individual samples can be mixed in any given pool. Each row of the tables corresponds to the parallel run of 96 tests on pools of the same size. The upper bound on the number of simultaneous tests comes from one of the typical sizes of commercially available multi-well plates where to run the tests. The maximum number of individual samples that can be mixed to detect the presence of a single one infected with SARS-CoV-2 using RT-qPCR has been estimated to be 32 in [38]. Pool overflow refers to the pools of the size listed in the row that come from those that tested positive at the previous stage which could not be tested because of lack of space in the multi-well plate. Table 1 corresponds to a situation in which the infected population is $p = 0.02$ for which the optimal number of stages for a scheme of the form $(3^k, \ldots, 3)$ is $k = k_3 = 3$. We then compare in this table the results of running the tests in parallel for the $(3^3, 3^2, 3)$ scheme and for a scheme that starts with $m_1 = 32$. We use the same population in both cases, with some added samples in the latter case. While the former scheme is optimal for $p = 0.02$ and satisfies that $m_1 < 32$, the latter puts the largest number of allowed individual samples in each of the first pools. Even if the former starts with fewer samples than the second one, it allows to resolve more cases after the first round of the strategy (first 4 time units in the table) because fewer pools (and samples) are left for the second round of stages. Furthermore, the number of tests per individual turns out to be smaller for the $(3^3, 3^2, 3)$ scheme. Changing the values of m_2 and m_3 in the scheme that starts with $m_1 = 32$ does not improve its performance. Namely, the case with $m_2 = 16$ and $m_3 = 4$ does not resolve all cases in two parallel rounds, but needs a third one. This is consistent with the proofs of [25] that we referred to before. As per the throughput time, if only the tests included in the table were run as listed in the table, the strategy that starts with $m_1 = 32$ samples would give a slightly better performance (384samples/unit-time) than the one that starts with $m_1 = 27$ (370samples/unit-time). If all 96 wells of the plate were used in each run, instead, the situation would be reversed, given that running the strategy with $m_1 = 27$ leaves many more empty wells in the second round (time units 5-7) where to accommodate additional pools than the one that starts with $m_1 = 32$. The analysis of how to optimize the use of the plates in detail is the matter of future work. We then perform the same comparison for $p = 0.008$ for which the optimal scheme of the form $(3^k, \ldots, 3)$ has $k = k_3 = 4$. In this case we would have to start with $m_1 = 2^4 = 81$ samples which is not allowed. Then, we compare the same two schemes as before and a scheme that starts with $m_1 = 32$ but has $k = 4$ instead of $k = 3$. We show the results in Table 2. There we see that there is no pool overflow in this case, something that we could have expected for the $(3^k, \ldots, 3)$ scheme based on the discussion of Fig. 4(b) in the main body of the paper. The comparison shows that the $(3^k, \ldots, 3)$ scheme is the one that requires the smallest number of tests per individual even if the number of stages is not the optimal for the infection probability of the population. That strategy is also the one that leaves the largest number of free wells where to accommodate new pools or samples to be tested among the two that are run in 4 time units.

Table 1. Comparison between the optimal strategy and one that starts with the maximum allowed number of samples in a pool.

$\mathbf{m_1 = 27}$, $\mathbf{m_2 = 9}$, $\mathbf{m_3 = 3}$, $\mathbf{m_4 = 1}$. **Samples: 2592. Infected: 53.**						
**Time Unit**	**Pool Size**	**# of Tests**	**Infected pools detected**	**Pool overflow**	**Identified Samples**	
					**Not-infected**	**Infected**
1	27	96	39	0	1539	0
2	9	96	41	21	495	0
3	3	96	35	27	183	0
4	1	96	32	9	64	32
**Accumulated Number of Cases Informed**					**2281**	**32**
5	9	21	8	0	117	0
6	3	51	18	0	99	0
7	1	63	21	0	42	21
**Accumulated Number of Cases Informed**					**2539**	**53**
**Tests/individual = 0.2002**						
$\mathbf{m_1 = 32}$, $\mathbf{m_2 = 8}$, $\mathbf{m_3 = 4}$, $\mathbf{m_4 = 1}$. **Samples: 3072. Infected: 64.**						
**Time Unit**	**Pool Size**	**# of Tests**	**Infected pools detected**	**Pool overflow**	**Identified Samples**	
					**Not-infected**	**Infected**
1	32	96	48	0	1536	0
2	8	96	29	96	536	0
3	4	58	30	0	112	0
4	1	96	25	24	71	0
**Accumulated Number of Cases Informed**					**2255**	**25**
5	8	96	31	0	520	0
6	4	62	32	0	120	0
7	1	96	25	56	71	25
**Accumulated Number of Cases Informed**					**2966**	**50**
8	1	56	14	0	42	14
**Accumulated Number of Cases Informed**					**3008**	**64**
**Tests/individual = 0.2135**						

Results of stochastic numerical simulations in which the nested pool strategy is performed simultaneously on up to 96 pools each of which can have at most 32 samples. At the first stage there are exactly 96 pools, but at the second stage the number can be larger. This is what we call “pool overflow” in the table. These unresolved pools can be tested whenever there is room. In the table we put in the same row all the pools of equal size that are available for testing at that point. The optimal strategy for $p = 0.02$ (the one with $m_1 = 27$) produces less overflow than the optimal one among those that start with the largest allowable number of samples in a pool ($m_1 = 32$). In this way, the strategy with $m_1 = 27$ finishes the processing of the 2592 initially tested samples in 7 time units while the one with $m_1 = 32$ requires 8 to process 3072.

Table 2. Comparison of strategies when the optimal includes pool sizes that are too large for a reliable detection.

$m_1 = 27$, $m_2 = 9$, $m_3 = 3$, $m_4 = 1$. Samples: 2592. Infected: 19.						
Time Unit	Pool Size	# of Tests	Infected pools detected	Pool overflow	Identified Samples Not-infected	Identified Samples Infected
1	27	96	15	0	2187	0
2	9	45	19	0	234	0
3	3	57	19	0	114	0
4	1	57	19	0	38	19
**Accumulated Number of Cases Informed**					**2573**	**19**
**Tests/individual = 0.0983**						

$m_1 = 32$, $m_2 = 8$, $m_3 = 4$, $m_4 = 1$. Samples: 3072. Infected: 25.						
Time Unit	Pool Size	# of Tests	Infected pools detected	Pool overflow	Identified Samples Not-infected	Identified Samples Infected
1	32	96	21	0	2400	0
2	8	84	24	0	480	0
3	4	48	24	0	96	0
4	1	96	25	0	71	25
**Accumulated Number of Cases Informed**					**3047**	**25**
**Tests/individual = 0.1055**						

$m_1 = 32$, $m_2 = 16$, $m_3 = 8$, $m_4 = 4$, $m_5 = 1$. Samples: 3072. Infected: 25.						
Time Unit	Pool Size	# of Tests	Infected pools detected	Pool overflow	Identified Samples Not-infected	Identified Samples Infected
1	32	96	21	0	2400	0
2	16	42	22	0	320	0
3	8	44	24	0	160	0
4	4	48	24	0	96	25
5	1	96	25	0	71	25
**Accumulated Number of Cases Informed**					**3047**	**25**
**Tests/individual = 0.1061**						

Similar to Table 1 but for a situation with a fraction of infected samples for which the optimal strategy requires that 81 samples be mixed together in the initial pools. In this case, the number of infected samples is low enough so that the average number of initial pools that test positive is very low and there is no "pool overflow".

References

[1] Bilder CR. In: Group Testing for Identification. American Cancer Society; 2019. p. 1–11. Available from: https://onlinelibrary.wiley.com/doi/abs/10.1002/9781118445112.stat08227.

[2] Bilder CR. In: Group Testing for Estimation. American Cancer Society; 2019. p. 1–11. Available from: https://onlinelibrary.wiley.com/doi/abs/10.1002/9781118445112.stat08231.

[3] Dorfman R. The Detection of Defective Members of Large Populations. Ann Math Statist. 1943;14(4):436–440. doi:10.1214/aoms/1177731363.

[4] Sterrett A. On the Detection of Defective Members of Large Populations. The Annals of Mathematical Statistics. 1957;28(4):1033–1036.

[5] Sobel M, Groll PA. Group testing to eliminate efficiently all defectives in a binomial sample. The Bell System Technical Journal. 1959;38(5):1179–1252. doi:10.1002/j.1538-7305.1959.tb03914.x.

[6] Hwang FK. A Method for Detecting All Defective Members in a Population by Group Testing. Journal of the American Statistical Association. 1972;67(339):605–608.

[7] Kotz S, Johnson NL. Errors in inspection and grading: distributional aspects of screening and hierarchal screening. Comm Statist A—Theory Methods. 1982;11(18):1997–2016. doi:10.1080/03610928208828366.

[8] Johnson NL, Kotz S, Wu XZ. Inspection errors for attributes in quality control. vol. 44 of Monographs on Statistics and Applied Probability. Chapman & Hall, London; 1991. Available from: https://doi.org/10.1007/978-1-4899-3196-2.

[9] Aldridge M. Rates of Adaptive Group Testing in the Linear Regime. 2019 IEEE International Symposium on Information Theory (ISIT). 2019; p. 236–240.

[10] Aldridge M, Johnson O, Scarlett J. Group Testing: An Information Theory Perspective. Foundations and Trends in Communications and Information Theory Series. Now Publishers; 2019. Available from: https://books.google.com.ar/books?id=7s5LzAEACAAJ.

[11] Phatarfod RM, Sudbury A. The use of a square array scheme in blood testing. Statistics in Medicine. 1994;13(22):2337–2343. doi:10.1002/sim.4780132205.

[12] Kim HY, Hudgens MG, Dreyfuss JM, Westreich DJ, Pilcher CD. Comparison of Group Testing Algorithms for Case Identification in the Presence of Test Error. Biometrics. 2007;63(4):1152–1163. doi:https://doi.org/10.1111/j.1541-0420.2007.00817.x.

[13] McMahan CS, Tebbs JM, Bilder CR. Two-Dimensional Informative Array Testing. Biometrics. 2012;68(3):793–804. doi:10.1111/j.1541-0420.2011.01726.x.

[14] Mézard M, Toninelli C. Group testing with random pools: optimal two-stage algorithms. IEEE Trans Inform Theory. 2011;57(3):1736–1745. doi:10.1109/TIT.2010.2103752.

[15] Hanel R, Thurner S. Boosting test-efficiency by pooled testing for SARS-CoV-2—Formula for optimal pool size. PLOS ONE. 2020;15(11):1–10. doi:10.1371/journal.pone.0240652.

[16] Mentus C, Romeo M, DiPaola C. Analysis and Applications of Non-Adaptive and Adaptive Group Testing Methods for COVID-19. medRxiv. 2020;doi:10.1101/2020.04.05.20050245.

[17] Sinnott-Armstrong N, Klein D, Hickey B. Evaluation of Group Testing for SARS-CoV-2 RNA. medRxiv. 2020;doi:10.1101/2020.03.27.20043968.

[18] Mutesa L, Ndishimye P, Butera Y, Souopgui J, Uwineza A, Rutayisire R, et al. A pooled testing strategy for identifying SARS-CoV-2 at low prevalence. Nature. 2020;doi:10.1038/s41586-020-2885-5.

[19] Mallapaty S. The mathematical strategy that could transform coronavirus testing. Nature. 2020;583:504 – 505. doi:10.1038/d41586-020-02053-6.

[20] Ben-Ami R, Klochendler A, Seidel M, Sido T, Gurel-Gurevich O, Yassour M, et al. Large-scale implementation of pooled RNA extraction and RT-PCR for SARS-CoV-2 detection. Clinical Microbiology and Infection. 2020;26(9):1248 – 1253. doi:https://doi.org/10.1016/j.cmi.2020.06.009.

[21] Shental N, Levy S, Wuvshet V, Skorniakov S, Shalem B, Ottolenghi A, et al. Efficient high-throughput SARS-CoV-2 testing to detect asymptomatic carriers. Science Advances. 2020;6(37). doi:10.1126/sciadv.abc5961.

[22] Barak N, Ben-Ami R, Sido T, Perri A, Shtoyer A, Rivkin M, et al. Lessons from applied large-scale pooling of 133,816 SARS-CoV-2 RT-PCR tests. medRxiv. 2020;doi:10.1101/2020.10.16.20213405.

[23] Suo T, Liu X, Feng J, Guo M, Hu W, Guo D, et al. ddPCR: a more accurate tool for SARS-CoV-2 detection in low viral load specimens. Emerging Microbes & Infections. 2020;9(1):1259–1268. doi:10.1080/22221751.2020.1772678.

[24] Pilcher CD, Westreich D, Hudgens MG. Group Testing for Severe Acute Respiratory Syndrome– Coronavirus 2 to Enable Rapid Scale-up of Testing and Real-Time Surveillance of Incidence. The Journal of Infectious Diseases. 2020;222(6):903–909. doi:10.1093/infdis/jiaa378.

[25] Armendáriz I, Ferrari PA, Fraiman D, Martínez JM, Dawson SP. Group testing with nested pools; 2020. https://arxiv.org/abs/2005.13650.

[26] Armendáriz I, Ferrari PA, Fraiman D, Martínez JM, Menzella HG, Sobral FNC, et al.. Pool testing calculator; 2021. http://www.pooling.df.uba.ar/.

[27] Black MS, Bilder CR, Tebbs JM. Optimal retesting configurations for hierarchical group testing. Journal of the Royal Statistical Society: Series C (Applied Statistics). 2015;64(4):693–710. doi:10.1111/rssc.12097.

[28] Yu F, Yan L, Wang N, Yang S, Wang L, Tang Y, et al. Quantitative Detection and Viral Load Analysis of SARS-CoV-2 in Infected Patients. Clinical Infectious Diseases. 2020;71(15):793–798. doi:10.1093/cid/ciaa345.

[29] Yang Q, Saldi TK, Gonzales PK, Lasda E, Decker CJ, Tat KL, et al. Just 2% of SARS-CoV-2-positive individuals carry 90% of the virus circulating in communities. Proceedings of the National Academy of Sciences. 2021;118(21). doi:10.1073/pnas.2104547118.

[30] Finucan HM. The Blood Testing Problem. Journal of the Royal Statistical Society Series C (Applied Statistics). 1964;13(1):43–50.

[31] Burrows P. Improved estimation of pathogen transmission rates by group testing. Phytopathology. 1987;77(5):363–365.

[32] Dube S, Qin J, Ramakrishnan R. Mathematical Analysis of Copy Number Variation in a DNA Sample Using Digital PCR on a Nanofluidic Device. PLOS ONE. 2008;3(8):1–9. doi:10.1371/journal.pone.0002876.

[33] Pan Y, Zhang D, Yang P, Poon LLM, Wang Q. Viral load of SARS-CoV-2 in clinical samples. The Lancet Infectious Diseases. 2020;20(4):411 – 412. doi:https://doi.org/10.1016/S1473-3099(20)30113-4.

[34] Zheng S, Fan J, Yu F, Feng B, Lou B, Zou Q, et al. Viral load dynamics and disease severity in patients infected with SARS-CoV-2 in Zhejiang province, China, January-March 2020: retrospective cohort study. BMJ. 2020;369. doi:10.1136/bmj.m1443.

[35] Liu X, Feng J, Zhang Q, Guo D, Zhang L, Suo T, et al. Analytical comparisons of SARS-COV-2 detection by qRT-PCR and ddPCR with multiple primer/probe sets. Emerging Microbes & Infections. 2020;9(1):1175–1179. doi:10.1080/22221751.2020.1772679.

[36] Sheskin DJ. Handbook of Parametric and Nonparametric Statistical Procedures. 5th ed. Chapman & Hall/CRC; 2011.

[37] Fleiss JL, Levin B, Paik MC. Statistical Methods for Rates and Proportions. 3rd ed. New York, NY John Wiley & Sons; 2003.

[38] Yelin I, Aharony N, Shaer-Tamar E, Argoetti A, Messer E, Berenbaum D, et al. Evaluation of COVID-19 RT-qPCR test in multi-sample pools. medRxiv. 2020;doi:10.1101/2020.03.26.20039438.
